# Bioengineering Insights into Orientation and Structural Stability of Phenyl Methyl Thiazole Derivative with β-Cyclodextrin Through Computational Modeling

**DOI:** 10.3390/bioengineering13050583

**Published:** 2026-05-19

**Authors:** Eswaran Kamaraj, Arumugam Anitha, Moorthiraman Murugan, Rajaram Rajamohan

**Affiliations:** 1 Department of Chemistry, Yeungnam University, Gyeongsan 38541, Republic of Korea; 2 PG and Research Department of Chemistry, Government Arts College, Chidambaram 608 102, Tamil Nadu, India; 3 Department of Chemistry, IFET College of Engineering, Villupuram 605 108, Tamil Nadu, India; 4 School of Chemical Engineering, Yeungnam University, Gyeongsan 38541, Republic of Korea

**Keywords:** *β*-cyclodextrin, inclusion complex, DFT, B3LYP-GD3/6-31G(d), complexation energy

## Abstract

This study explores the formation of inclusion complexes between a newly synthesized N-(2-(butylamino)-2-oxoethyl)-2-(3-cyano-4-isobutoxyphenyl)-4-methylthiazole-5-carboxamide with β-cyclodextrin using density functional theory with dispersion correction (DFT-D3) at the B3LYP-GD3/3-21G, 6-31G(d), 6-31G’(d), and 6-311G(d) levels. Two orientations are considered: in Orientation A, the 3-cyano-4-isobutoxyphenyl moiety interacts with the primary hydroxyl rim of β-cyclodextrin, while in Orientation B, the amide side chain faces the wider rim. Complexation energies and thermodynamic parameters are calculated to determine stability. Electronic properties, including HOMO-LUMO energies, and global reactivity descriptors, such as electronegativity (χ), chemical potential (μ), hardness (η), and electrophilicity index (ω), are evaluated. Non-covalent interaction (NCI) analysis is also performed to visualize interaction sites. The results reveal the significant influence of orientation on the host–guest complex stability and electronic properties, providing valuable insights into cyclodextrin-based encapsulation systems. The study provides a computational blueprint for engineering cyclodextrin-based bio-functional systems, where orientation-controlled inclusion governs stability, reactivity, and performance. This can significantly impact the development of smart drug delivery systems, biosensors, and multifunctional biomaterials in modern bioengineering.

## 1. Introduction

The demand for newly synthesized organic drug molecules exhibiting promising biological activity is ever-growing. However, these molecules often face significant solubility, stability, and biocompatibility challenges, among other limitations [1,2]. Addressing these drawbacks is critical to enhancing the efficacy and applicability of these drugs. Numerous strategies have been developed to overcome these issues, with supramolecular assembly involving cyclodextrins (CDs) emerging as a highly effective approach [3,4,5,6]. This method offers significant advantages, including improved solubility, stability, and biocompatibility, without altering the inherent chemical properties of the drug molecules. CDs are cyclic oligosaccharides composed of glucose units linked via α-1,4-glycosidic bonds, forming a toroidal structure. Common types of CDs, such as α-, β-, and γ-CDs, are widely utilized in supramolecular chemistry due to their ability to form inclusion complexes (ICs) with a diverse range of guest molecules [4,5,6]. This capability is rooted in their unique structural features: a hydrophilic outer surface and a hydrophobic inner cavity. These structural characteristics enable CDs to interact non-covalently with guest molecules, enhancing their solubility, stability, and bioavailability. Consequently, CDs and their inclusion complexes have become indispensable in various pharmaceutical and industrial applications. Despite their benefits, understanding the formation, stability, and functional properties of CD-based ICs presents considerable challenges due to the dynamic and intricate nature of host–guest interactions. This complexity necessitates advanced computational methodologies to complement experimental techniques. Computational approaches offer a cost-effective and time-efficient means of investigating the orientation, stability, and molecular interactions within ICs.

In traditional experimental (wet lab) methods, researchers can analyze the orientation and stability of ICs within the CD cavity and its derivatives. This foundational understanding enables the development of optimal conditions for complexation, which can then be validated through further experimental techniques. Once the ICs demonstrate the desired stability and orientation, they can be subjected to biological evaluation and tested for their suitability in drug delivery applications. Density functional theory (DFT), a quantum mechanical computational technique, has proven to be an essential tool in studying CD-based ICs [7,8]. DFT allows researchers to delve into the electronic properties, interaction mechanisms, and stability of host–guest systems at the atomic level. By quantifying molecular interactions and providing insights into the electronic structure of ICs, DFT facilitates the design and optimization of drug delivery systems, reducing dependency on exhaustive trial-and-error experimental approaches [9,10]. In recent years, numerous studies have employed DFT as a computational approach to investigate the molecular interactions between drug molecules and CDs. These investigations have focused on understanding key aspects such as binding energies, interaction mechanisms, and the stability of the resulting inclusion complexes [11,12,13,14].

**Figure 1:**
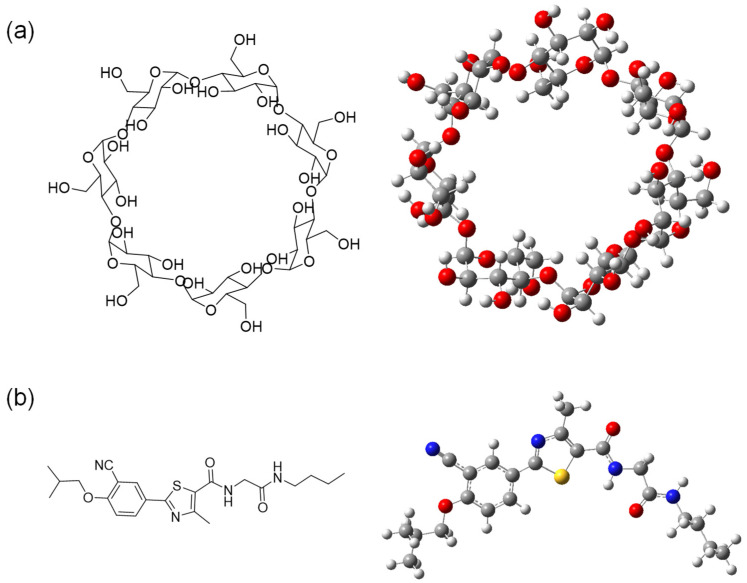
Chemical structure and the minimized 3D shape of β-CD (**a**) and PMT (**b**).

The objective of the present study is to explore the viability of forming inclusion complexes (ICs) between a newly synthesized phenyl methyl thiazole derivative (PMT) and the cavity of β-cyclodextrin (β-CD). The detailed chemical structures and 3D shape of β-CD and PMT are shown in Figure 1. As reported in previous studies, this compound has already demonstrated promising biological activities, including anti-inflammatory and anti-diabetic effects [15]. A detailed computational investigation is essential to complement the already established experimental findings on the interaction between the drug compound, PMT, and the related structures with β-CD [16,17]. Such theoretical analyses provide deeper molecular-level insights into the stability, preferred orientation, binding affinity, and thermodynamic feasibility of the inclusion complex, thereby supporting and rationalizing the experimentally observed improvements in solubility, stability, and biological activity. Furthermore, computational studies help elucidate the nature of the host–guest interactions within the β-CD cavity, offering a clearer understanding of the encapsulation mechanism and the factors governing complex stability.

This research analyzes the interaction between the compound and β-CD using a DFT approach with an appropriate basis set. Specifically, the DFT method, including dispersion correction (DFT-D3), was utilized to study the ICs in the gas phase, aiming to determine the nature of the host–guest interaction and identify energetically favorable binding orientations. Additionally, non-covalent interaction analysis was performed to characterize the nature of these interactions further. Furthermore, the study evaluates the thermodynamic properties of the ICs, providing insights into their stability and possibility for practical applications. These findings will serve as a foundation for subsequent experimental methodologies and biological evaluations.

## 2. Results and Discussion

### 2.1. Computational Approach

The inclusion complexes (ICs) of β-cyclodextrin (β-CD) with PMT are comprehensively studied using DFT calculations performed at the B3LYP-GD3/6-31G(d) levels of theory to ensure a comprehensive analysis of their electronic properties and interaction strengths. Two distinct orientations of the PMT molecule within the β-CD cavity, designated as Ori-1 and Ori-2, are examined to assess their structural and electronic characteristics. The analysis focused on the optimized geometries of these complexes, providing insights into their spatial arrangement and molecular interactions. Additionally, the study explored the electronic properties by evaluating the frontier molecular orbitals (FMOs), including the highest occupied molecular orbital (HOMO) and lowest unoccupied molecular orbital (LUMO), which are critical for understanding electronic transitions and chemical stability. Furthermore, global reactivity descriptors, such as chemical potential, hardness, softness, and electrophilicity, are derived from the calculated orbital energies to elucidate the reactivity and stability of the inclusion complexes in each orientation. This section details the findings from these calculations, highlighting the comparative structural and electronic features of Ori-1 and Ori-2 configurations.

### 2.2. Complexation Energy Analysis

The stability of the β-CD and PMT ICs was systematically evaluated by calculating their complexation energies, providing a quantitative measure of the binding strength between the host (β-CD) and the guest molecules in two distinct orientations. These calculations offer valuable insights into the thermodynamic favorability of the host–guest interactions [18]. The complexation energies were determined using the B3LYP-GD3 functional with the 6-31G(d) basis set, incorporating explicit BSSE corrections to ensure accuracy.

For the Ori-1 orientation, the complexation energy was calculated as −10.96 kcal/mol, indicating a moderately stable interaction between β-CD and PMT. While the binding is favorable, it is relatively weaker than that of other configurations. In contrast, the Ori-2 orientation exhibits a significantly more negative complexation energy of −41.49 kcal/mol, suggesting a much stronger and more thermodynamically favorable binding interaction. This pronounced difference highlights the enhanced stabilization achieved in the Ori-2 configuration. These findings underscore the critical influence of molecular orientation on host–guest stabilization. The consistently negative complexation energy for Ori-2 reinforces its greater stability compared to Ori-1, aligning with trends observed in electronic properties and reactivity descriptors. This study deepens our understanding of the molecular factors governing β-CD and PMT IC stability and emphasizes the importance of selecting an appropriate computational approach to accurately capture the intricacies of host–guest interactions.

### 2.3. Structural and Electronic Properties

**Figure 2:**
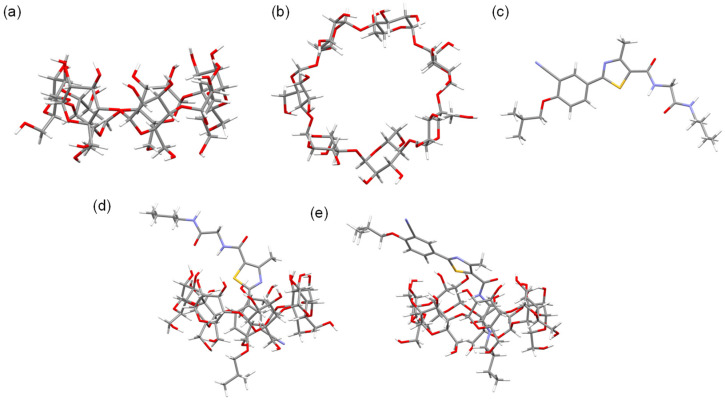
DFT/B3LYP-GD3/6-31G(d) optimized structures of (**a**,**b**) β-CD, (**c**) PMT, (**d**) PMT:β-CD ICs Ori-1 and (**e**) PMT:β-CD ICs Ori-2.

**Figure 3:**
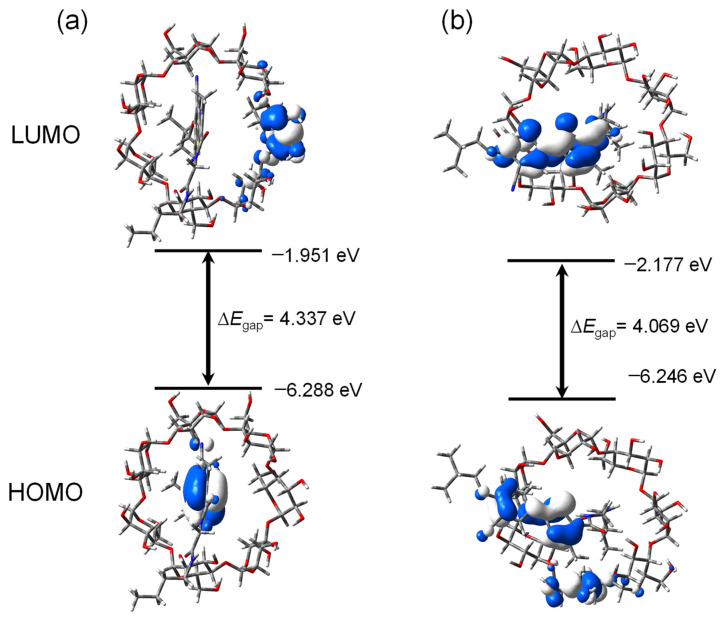
FMO diagram, HOMO & LUMO energy values along with the energy gaps of (**a**) PMT:β-CD ICs Ori-1 and (**b**) PMT:β-CD ICs Ori-2 determined by the DFT/B3LYP-GD3/6-31G(d) method.

The optimized geometries of the β-CD and PMT ICs are depicted in Figure 2, highlighting the spatial arrangement of the PMT molecule within the β-CD cavity for both orientations, Ori-1 and Ori-2. These configurations are characterized by their stability, with the PMT molecule securely embedded in the β-CD cavity. Structural analysis reveals notable differences between Ori-1 and Ori-2 in terms of binding interactions and spatial alignment, which in turn influence their electronic properties. The energy levels of the FMOs, including the HOMOs and the LUMOs, for β-CD, PMT, Ori-1, and Ori-2, are presented in Table 1. Figure 3 provides a visual representation of the FMO distributions, showing the spatial localization of the HOMO and LUMO orbitals and indicating the regions most likely to engage in electron donation or acceptance.

As the host molecule, β-CD demonstrates the highest energy gap (ΔE = 5.7 eV), underscoring its intrinsic stability and reduced chemical reactivity. In contrast, PMT, the guest molecule, has a lower energy gap of 4.126 eV, signifying increased chemical reactivity due to its more accessible frontier orbitals. The formation of ICs between β-CD and PMT results in a further reduction in the energy gaps, indicating changes in the electronic structure upon host–guest complex formation [18]. For the Ori-1 configuration, the energy gap (ΔE) is calculated as 4.337 eV, indicating moderate reactivity. The Ori-2 configuration, however, exhibits a slightly smaller energy gap of 4.069 eV, suggesting comparatively higher electronic polarizability and reactivity. This difference between Ori-1 and Ori-2 may be associated with variations in host–guest orientation and electronic interactions within the complexes. However, the HOMO-LUMO gap mainly reflects the electronic characteristics of the inclusion complexes and does not directly account for dominant dispersion-driven interaction governing β-CD host–guest complexation.

### 2.4. Non-Covalent Interaction (NCI) Analysis

To gain deeper insights into the non-covalent interactions governing the inclusion complexes of PMT and β-CD, an NCI analysis based on the Reduced Density Gradient (RDG) method was performed. This approach enables visualization and characterization of weak intermolecular forces, including van der Waals interactions, hydrogen bonding, and steric repulsion.

**Figure 4:**
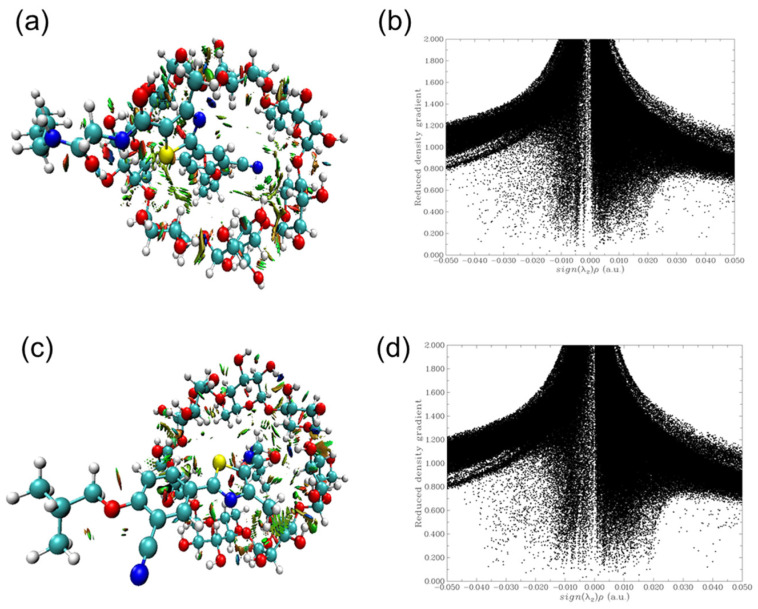
NCI isosurfaces and NCI-RDG scatter plots of PMT:β-CD (**a**,**b**) Ori-1 and (**c**,**d**) Ori-2 based on B3LYP-GB3/6-31G(d) calculations.

Figure 4a,b correspond to Ori-1, while Figure 4c,d represent Ori-2. The NCI isosurfaces (Figure 4a,c) illustrate the spatial distribution of weak interactions within the host–guest complexes. The corresponding scatter plots (Figure 4b,d) depict the RDG as a function of the electron density multiplied by the sign of the second Hessian eigenvalue (sign(λ_2_)ρ), spanning a range from −0.05 to 0.05 a.u. The NCI-RDG analysis reveals distinct interaction patterns for the two orientations. In both complexes, green regions on the iso-surfaces indicate the presence of weak van der Waals interactions, which play a significant role in stabilizing the ICs. Blue regions represent strong hydrogen bonding interactions, highlighting key stabilization sites where PMT interacts with β-CD through hydrogen donor–acceptor pairs. Conversely, red regions correspond to steric repulsion, indicating regions of spatial strain or unfavorable overlap between electron clouds. A comparative analysis of Ori-1 and Ori-2 suggests notable differences in interaction strength and spatial distribution. The Ori-2 complex (Figure 4c,d) exhibits a higher concentration of blue regions, signifying a greater degree of hydrogen bonding and stronger attractive forces compared to Ori-1. Additionally, the scatter plot for Ori-2 (Figure 4d) shows a more pronounced density of negative sign(λ_2_)ρ values, further confirming the enhanced stabilization through electrostatic and hydrogen bonding interactions. These findings corroborate the energetic analysis, reinforcing the conclusion that Ori-2 is the thermodynamically and chemically preferred orientation for complex formation. The greater abundance of stabilizing non-covalent interactions in Ori-2 underpins its superior binding affinity and enhanced structural stability, aligning with the observed trends in complexation energy and electronic properties.

### 2.5. Global Reactivity Descriptors

The global reactivity descriptors chemical potential (μ), electronegativity (χ), hardness (η), softness (S), and electrophilicity index (ω) offer a comprehensive understanding of the reactivity and stability of the β-CD and PMT inclusion complexes [19,20]. The calculated values, presented in Table 2, reveal distinct trends and provide valuable insights into the properties of the studied systems. Among the configurations, Ori-2 exhibits the lowest chemical potential (μ = −4.2115 eV), signifying its superior thermodynamic stability compared to Ori-1 and the individual components, β-CD and PMT. This enhanced stability is indicative of stronger host–guest interactions in the Ori-2 orientation. The higher electronegativity values of Ori-1 (χ = 4.1195 eV) and Ori-2 (χ = 4.2115 eV) relative to β-CD and PMT highlight the increased charge transfer capabilities of the inclusion complexes. These elevated χ values align with the observed strong binding interactions within the host–guest systems. The hardness (η) and softness (S) parameters further emphasize the differences in stability and reactivity among the systems. Ori-2, with the smallest hardness (η = 2.0345 eV) and the highest softness (S = 1.0172 eV), is identified as the most reactive configuration. Its softer nature makes Ori-2 more adaptable to electronic interactions, facilitating effective complexation with PMT. In contrast, the higher hardness and lower softness of β-CD and PMT suggest reduced adaptability and reactivity in their isolated forms. The electrophilicity index (ω) provides an additional layer of differentiation between the systems. Ori-2 displays the highest electrophilicity value (ω = 4.358), followed by Ori-1 (ω = 3.913), PMT (ω = 3.711), and β-CD (ω = 2.400). The elevated electrophilicity of Ori-2 underscores its superior electronic-accepting ability, which may contribute to favorable electronic interactions within the complex.

It should be noted that the HOMO-LUMO gap and global reactivity descriptor mainly reflect the electronic characteristics and relative electronic stabilization of the complexes. Theses descriptors do not directly account for the dispersion and van der Walls interactions that predominantly govern CD host–guest ICs. Therefore, the enhanced stability of Ori-2 is more appropriately attributed to the combined effects of geometric fitting, hydrogen binding, van der Walls forces and dispersion-driven non-covalent interactions, while the global descriptors provide complementary insight into the electronic aspects of complex formation.

### 2.6. Implications

The electronic properties revealed in this study shed light on the fundamental mechanisms driving inclusion complexation. In its free state, β-CD displays a large energy gap (ΔE) indicative of its inherent stability and low reactivity. However, upon forming complexes with PMT, both Ori-1 and Ori-2 exhibit marked reductions in their energy gaps, signifying enhanced reactivity as a result of robust host–guest interactions. Among the two configurations, Ori-2 stands out as the most reactive and electrophilic. Its notably lower energy gap, increased electronegativity, and greater softness suggest a heightened ability for binding and charge transfer, implying that Ori-2 establishes a stronger and more stable complex with PMT.

Further supporting these observations, global reactivity descriptors indicate that Ori-2 exhibits a higher chemical potential, lower hardness, and elevated electrophilicity compared to Ori-1, reinforcing its superior adaptability in facilitating host–guest interactions. The orientation of the guest molecule plays a crucial role in determining these properties, significantly influencing the stability and binding efficiency of the inclusion complex. With BSSE-corrected complexation energies, Ori-1 demonstrates a moderately stable interaction with a binding energy of −10.96 kcal/mol. In contrast, the Ori-2 configuration exhibits a substantially more negative complexation energy of −41.49 kcal/mol, emphasizing its stronger and more thermodynamically favorable binding interaction. This pronounced difference underscores the critical influence of molecular orientation on the stability and electronic properties of the inclusion complexes [21]. These results further validate the preferential stability of the Ori-2 configuration and provide a strong theoretical foundation for future experimental investigations. The findings offer valuable insights into host–guest chemistry, particularly in designing ICs with optimized binding and functional properties.

### 2.7. Hydrogen Bonding Interactions

**Figure 5:**
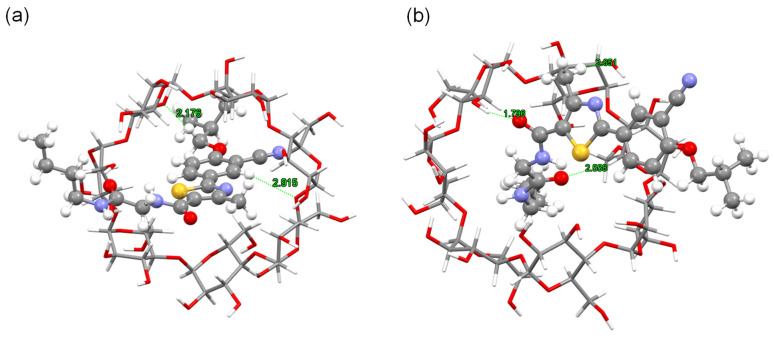
Hydrogen bonding in PMT:β-CD ICs with (**a**) Ori-1 and (**b**) Ori-2 at B3LYP-GD3/6-31G(d) method.

The hydrogen bonding interactions between β-CD and PMT play a critical role in stabilizing their ICs, as detailed in Figure 5 and Table 2 [22]. These interactions reveal distinct patterns in the two orientations, Ori-1 and Ori-2, which significantly influence the stability and structural properties of the complexes.

#### 2.7.1. ICs with Ori-1

In the ICs with Ori-1, several key hydrogen bonds are observed between β-CD and PMT, contributing to the stability of the complex: (i) C_1_-O and C_18_ Interaction: A significant hydrogen bond forms between the carbonyl oxygen (C_1_-O) of β-CD and the C_18_ position of PMT, with a bond length of 2.915 Å. This interaction is indicative of strong stabilization. (ii) C_6_ Hydroxyl Group: The hydroxyl group at β-CD’s C_6_ forms a hydrogen bond with the C_4_ position of PMT, with a bond length of 2.176 Å. A second hydrogen bond forms between β-CD’s C_6_ hydroxyl group and PMT’s C_10_, with a bond length of 2.393 Å. These interactions highlight the importance of the C_6_ position in coordinating with the functional groups of PMT. (iii) C_6_-O and C_21_ Interaction: The oxygen atom at β-CD’s C_6_ interacts with the C_21_ group of PMT, with a bond length of 2.616 Å, further stabilizing the complex. (iv) C_5_-O and Amide Group (HN-C_17_): A hydrogen bond forms between the C_5_-O group on β-CD and the amide group of PMT (HN-C_17_), with a bond length of 2.162 Å, reinforcing the role of exterior hydroxyl groups in stabilizing the complex. These interactions collectively demonstrate that the hydroxyl groups at the C_1_ and C_6_ positions of β-CD are pivotal in facilitating the inclusion of PMT. The combination of direct hydrogen bonding and electrostatic interactions creates a stable and structured host–guest complex.

#### 2.7.2. ICs with Ori-2

The ICs with Ori-2 exhibit a distinct pattern of hydrogen bonding interactions, predominantly involving β-CD’s C_2_ and C_3_ hydroxyl groups. Key interactions include: (v) C_2_-O and Methyl Group (H-C_14_): A hydrogen bond forms between β-CD’s C_2_-O and the methyl group (H-C_14_) on PMT, with a bond length of 2.557 Å, stabilizing the guest molecule in this orientation. (vi) C_3_-OH and Carbonyl Oxygen (O-C_15_): A particularly strong hydrogen bond is observed between β-CD’s C_3_ hydroxyl group and the carbonyl oxygen of PMT, with a bond length of 1.796 Å. The short distance suggests this is one of the strongest stabilizing interactions in the complex. (vii) C_5_-O and Amide Group (HN-C_17_): Similar to Ori-1, a hydrogen bond forms between the C_5_-O group on β-CD and the amide group of PMT (HN-C_17_), with a bond length of 2.162 Å. (viii) C_6_-H and Nitrile Nitrogen (N-C_10_): A hydrogen bond is observed between the C_6_-H group of β-CD and the nitrile nitrogen of PMT, with a bond length of 2.393 Å, enhancing the overall stability of the complex. These interactions highlight that β-CD’s C_2_, C_3_, and C_6_ positions are critical in Ori-2, establishing a network of hydrogen bonds with PMT’s functional groups. The shorter bond lengths, particularly the C_3_-OH interaction with the carbonyl oxygen, suggest stronger binding and a more stable IC.

#### 2.7.3. Comparison of Ori-1 and Ori-2

The primary difference between the two configurations lies in the specific hydrogen bonding interactions: (i) Ori-1: Stabilization is driven by interactions involving β-CD’s C_1_ and C_6_ positions, with significant contributions from the hydroxyl and carbonyl groups. (ii) Ori-2: The C_2_ and C_3_ hydroxyl groups of β-CD dominate the interaction, forming stronger hydrogen bonds with PMT’s carbonyl and amide groups. These variations result in unique structural characteristics and stability profiles for each complex. The stronger hydrogen bonding network in Ori-2 explains its greater thermodynamic stability and preference as the energetically favored orientation, as supported by complexation energy and global reactivity descriptor analyses.

### 2.8. Thermodynamic Analysis of PMT:β-CD ICs

The thermodynamic parameters for the PMT:β-CD ICs with Ori-1 and Ori-2 are calculated to evaluate the stability and spontaneity of the complexation process [23,24]. These parameters, including enthalpy change (∆H), Gibbs free energy change (∆G), and entropy change (∆S), provide valuable insights into the energetic favorability and molecular interactions underlying the formation of the complexes.

#### 2.8.1. Enthalpy Change (∆H)

The enthalpy change for the formation of PMT:β-CD ICs Ori-1 is calculated as −33.64 kcal/mol, indicating an exothermic process. This negative ∆H suggests that the formation of Ori-1 is energetically favorable, driven primarily by the release of energy during host–guest interactions such as hydrogen bonding and van der Waals forces. For PMT:β-CD ICs Ori-2, the enthalpy change is even more negative at −38.22 kcal/mol, signifying a stronger exothermic interaction compared to Ori-1. This greater release of energy indicates stronger binding interactions and enhanced stability in Ori-2. The larger magnitude of ∆H for Ori-2 highlights the significant contribution of its unique hydrogen bonding network and optimized spatial arrangement to the overall stabilization of the system.

#### 2.8.2. Gibbs Free Energy Change (∆G)

The Gibbs free energy change (∆G) for PMT:β-CD ICs Ori-1 is 3.38 kcal/mol. The positive value indicates that the formation of Ori-1 is not spontaneous under standard conditions, despite being exothermic. This result suggests that additional energy or favorable conditions (e.g., higher temperatures or specific environmental factors) might be required to drive the complexation process for Ori-1. In contrast, the ∆G for PMT:β-CD ICs Ori-2 is −15.06 kcal/mol. The negative value clearly demonstrates that the formation of Ori-2 is spontaneous and thermodynamically favorable. This finding reinforces the superior stability and binding efficiency of the Ori-2 orientation, consistent with its more negative enthalpy change and stronger hydrogen bonding network.

#### 2.8.3. Entropy Change (∆S)

The entropy change (∆S) is negative for both complexes, reflecting the ordering of the system during complexation. For PMT:β-CD IC Ori-1, ∆S is −0.5196 J/mol·K, while for Ori-2, ∆S is slightly less negative at −0.3250 J/mol·K. The negative entropy values indicate that the formation of the ICs leads to increased structural organization, likely due to the constrained movement of PMT within the β-CD cavity and the induced rigidity in β-CD upon complexation. The smaller magnitude of ∆S for Ori-2 compared to Ori-1 suggests that the formation of Ori-2 induces slightly less loss of entropy, potentially due to a more efficient fit of PMT into the β-CD cavity. This reduced entropy penalty, coupled with the highly exothermic enthalpy change, contributes to the overall spontaneity and enhanced stability of Ori-2.

#### 2.8.4. Comparison and Implications

The thermodynamic analysis reveals that while both ICs with Ori-1 and Ori-2 are energetically favorable (negative ∆H), only Ori-2 exhibits spontaneous formation under standard conditions (negative ∆G). The stronger exothermic interaction and reduced entropy penalty in Ori-2 underscore its enhanced thermodynamic stability and preference for complexation. These findings align with the observed electronic properties, hydrogen bonding patterns, and complexation energy calculations, collectively highlighting Ori-2 as the energetically and thermodynamically favored configuration. The results provide critical insights for understanding the inclusion mechanism and optimizing host–guest systems for potential applications in drug delivery, catalysis, and molecular encapsulation.

#### 2.8.5. Comparison of Computer Modeling with Experimental Results

The experimental and computational investigations collectively provide strong evidence for the successful formation and enhanced performance of the IC between the guest molecule and β-CD. Experimentally, the PMT:β-CD system demonstrated improved aqueous solubility, enhanced bioavailability, and significantly increased anti-inflammatory activity compared with the free drug molecule [16,17]. Spectroscopic characterization, including FT-IR, NMR, and ROESY analyses, confirmed the formation of a stable 1:1 host–guest complex, where the amide and ethyl moieties of PMT were preferentially accommodated within the hydrophobic cavity of β-CD. The enhanced therapeutic response without noticeable toxicity further indicated that encapsulation within β-CD improved the pharmacological profile of the guest molecule while maintaining biocompatibility.

The computational analysis strongly supports these experimental observations by elucidating the molecular-level interactions responsible for the stability of the inclusion complex. DFT calculations revealed that both orientations (Ori-1 and Ori-2) can form stable host–guest assemblies; however, Ori-2 was identified as the most favorable configuration. The more negative complexation energy, lower HOMO–LUMO energy gap, higher electrophilicity, and increased softness of Ori-2 indicate stronger host–guest interactions and greater chemical reactivity, which are consistent with the experimentally observed enhancement in solubility and biological activity. Furthermore, non-covalent interaction analysis demonstrated stronger hydrogen bonding interactions in Ori-2, whereas Ori-1 exhibited comparatively higher steric repulsion, explaining the preferential stabilization of the Ori-2 geometry within the β-CD cavity.

Importantly, the computational thermodynamic analysis aligns well with the experimental findings. The negative Gibbs free energy (ΔG) calculated for Ori-2 confirms the spontaneous and thermodynamically favorable nature of the complexation process, correlating with the experimentally verified stable encapsulation and improved physicochemical behavior of the IC. In contrast, the thermodynamically unfavorable Ori-1 orientation suggests that not all possible insertion modes contribute equally to complex stability. This agrees with the ROESY and NMR observations, which indicated a preferred orientation of the guest molecule inside the β-CD cavity. 

## 3. Conclusions

The computational work presented here demonstrates the interaction between the guest PMT and host β-CD using a dispersion-corrected density functional theory (DFT) approach incorporating the 6-31G(d) basis set, confirming that both Ori-1 and Ori-2 can form stable ICs. However, Ori-2 exhibits superior reactivity and binding properties, as demonstrated by its more negative complexation energy, lower energy gap, higher electrophilicity, and greater softness across the entire basis set. Non-covalent interaction analysis further supports these findings, demonstrating that Ori-2 exhibits stronger hydrogen bonding interactions, whereas Ori-1 shows greater steric repulsion. This suggests that Ori-2 provides a more stable encapsulation environment for PMT within the β-CD cavity. Moreover, free energy calculation indicates that only Ori-2 exhibits a negative ∆G, confirming its thermodynamic feasibility, whereas Ori-1 remains thermodynamically unfavorable despite its exothermic enthalpy change. This highlights the superior stability and spontaneity of the Ori-2 complexation, further reinforcing its potential for practical applications.

## 4. Future Research Directions

There are several promising bioengineering-focused future directions emerging from this research. Some of them are listed as follows: (i) Molecular dynamics-guided optimization for biomolecular environments: Perform MD simulations to gain deeper insights into the dynamic behavior, stability, and intermolecular interactions of Ori-1 and Ori-2 ICs under physiologically relevant solvent environments. Such studies can help elucidate solvation effects, conformational flexibility, and molecular recognition mechanisms, supporting the rational engineering of CD-based carriers for responsive drug delivery, biomolecular transport, and nano-bio interfaces. (ii) Engineering host–guest interactions for functional biomaterials: Investigate specific host–guest interaction mechanisms using quantum chemical calculations, focusing on hydrogen bonding, van der Waals interactions, and π–π stacking to identify the dominant contributors to the superior binding properties of Ori-2. These insights can support the molecular engineering of optimized supramolecular architectures for bioactive encapsulation, controlled release platforms, biosensing systems, and functional biomaterials. (iii) Translation toward advanced drug delivery and biomedical applications: Evaluate the potential of Ori-2/β-CD ICs for bioengineering applications in drug delivery systems, particularly their ability to enhance the solubility, stability, bioavailability, and controlled release of active pharmaceutical ingredients. Future studies may extend toward nanocarrier fabrication, targeted delivery systems, tissue engineering scaffolds, and stimuli-responsive therapeutic platforms. (iv) Integration into emerging bioengineering technologies: Future research may also explore integrating these ICs into advanced bioengineering platforms, including nanomedicine, bioresponsive hydrogels, implantable delivery devices, and multifunctional therapeutic systems. Such efforts could broaden the practical utilization of CD-based ICs in translational biomedical engineering. By addressing these directions, future research can build upon the foundational insights of this study and advance the development of supramolecular bioengineering systems based on β-CD for diverse pharmaceutical and biomedical applications.

## 5. Methods

The molecular modeling of the inclusion complex between β-CD and the guest molecule PMT was initiated by obtaining the initial molecular structure of β-CD from its corresponding crystallographic data. This approach ensured a high degree of structural accuracy and fidelity in the host model. The PMT molecule was constructed de novo and subsequently optimized using HyperChem 8.0, a robust software system for molecular modeling and geometry optimization. To investigate the interaction between β-CD and PMT, advanced computational analyses were carried out using the Gaussian 16W program suite [25]. This suite was chosen for its reliability and versatility in handling molecular interaction studies, particularly for host–guest systems. The geometries of both the individual molecules and their inclusion complexes were optimized using density functional theory (DFT). DFT is widely regarded as a precise and computationally efficient method for probing molecular structures and interactions.

For this study, B3LYP (Becke’s three-parameter exchange functional with the Lee–Yang–Parr correlation functional) was chosen, particularly the B3LYP-GD3 functional [26]. This function, which incorporates Grimme’s D3 dispersion correction, was chosen due to its enhanced ability to model non-covalent interactions and recognized performance in studying CD-based ICs, offering a good balance between accuracy and computational cost. B3LYP has been successfully used in previous studies to describe hydrogen bonding, dispersion interactions, and electrostatic effects in similar host–guest systems. While more advanced functionals (e.g., M06-2X) may offer improved dispersion modeling, B3LYP remains a standard benchmark functional for CD ICs and has been validated against experimental findings in previous research [27,28].

To ensure a comprehensive and comparative analysis of the ICs, multiple basis sets were considered. The 6-31G(d) basis set was employed as a cost-effective approach for initial screening, providing a reliable estimation of structural and energetic properties. Additionally, the inclusion of polarization functions in the 6-31G(d) basis set enhances the accuracy of non-covalent interaction descriptions, particularly for hydrogen bonding and van der Waals forces, which are crucial in host–guest interactions [29]. This methodological approach ensures a balanced trade-off between computational efficiency and accuracy in capturing the key interactions governing the stability of the ICs.

**Figure 6:**
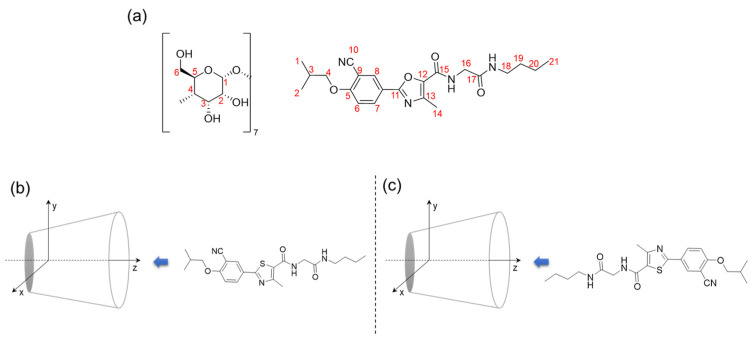
(**a**) Molecular structure of β-CD & PMT with numbering; graphical representation of possible PMT:β-CD ICs, (**b**) Ori-1 and (**c**) Ori-2.

The process of complexation was analyzed using a defined coordinate system, which provided a systematic framework for interpreting the spatial orientation and interaction dynamics of the inclusion complexes. As illustrated in Figure 6, two potential orientations of the PMT molecule within the β-CD cavity were considered for analysis. These orientations represent plausible configurations based on steric and electronic compatibility and were subjected to the same rigorous optimization process to evaluate their structural and energetic properties comprehensively. This modeling approach ensured an in-depth understanding of the interaction between β-CD and PMT, paving the way for further exploration of their inclusion properties. Orientation 1 (Ori-1): The aromatic part of the PMT molecule faces the secondary hydroxyl groups of β-CD. Orientation 2 (Ori-2): The amide chain of PMT is directed towards the secondary hydroxyl groups of β-CD.

To establish the coordination system, the glycosidic oxygen atoms of the β-CD molecule were positioned on the XY plane, and their geometric center was set as the origin of the coordinate system. Subsequently, the PMT molecule was incrementally moved into the β-CD cavity along the *Z*-axis to simulate the complexation process. Finally, the binding energy (∆E) of the β-CD–PMT complex was calculated for the optimized minimum-energy structures using the following equation (Equation (1)), which quantifies the interaction energy upon complex formation [30].

(1) $$∆E=E_{PMT:\beta CD}-\left(E_{\beta CD}+E_{PMT}\right)+BSSE$$

where *E*_PMT/βCD_ represents the total energy of the PMT:β-CD ICs and *E*_βCD_ and *E*_PMT_ denote the energies of isolated *β*-CD and PMT molecules, respectively. To account for the basis set’s incompleteness in binding energy calculations, the basis set superposition error (BSSE) was incorporated using the counterpoise method proposed by Boys and Bernadi [31,32]. This equation quantifies the binding energy by subtracting the sum of the individual components’ energies from the energy of the complex, providing a measure of the stability and strength of the interaction between β-CD and PMT.

To further characterize the nature of intermolecular interactions, non-covalent interaction (NCI) analysis was conducted using the Reduced Density Gradient (RDG) method, which provides a detailed visualization of weak interactions, including hydrogen bonding, van der Waals forces, and steric effects [33]. The RDG calculations were performed based on the B3LYP-GD3/6-31G(d) optimized geometries, ensuring high accuracy in capturing the interaction regions. The obtained electron density and Reduced Density Gradient data were analyzed using VMD 1.9.1 (Visual Molecular Dynamics) software to generate NCI-RDG isosurfaces, where green regions represent weak van der Waals interactions, blue regions indicate strong attractive hydrogen bonding, and red regions correspond to steric repulsive interactions [34]. Additionally, scatter plots of sign(λ_2_)ρ versus RDG were plotted to quantitatively assess the nature and strength of the interactions. These analyses provided a detailed visualization of the binding environment in both Ori-1 and Ori-2 configurations, offering deeper insights into host–guest stabilization mechanisms. The integration of DFT-based energy calculations with NCI-RDG analysis ensures a comprehensive understanding of the electronic properties, interaction strength, and stability of β-CD:PMT ICs.

The thermodynamic parameters for the inclusion complexes were calculated by obtaining the enthalpy (∆H), entropy (∆S), and Gibbs free energy (∆G) using the following formulas [35]. Enthalpy (∆H) was calculated using the difference in the total energies of the system at the optimized geometry. Entropy (∆S) was derived from the difference in the total entropy of the system between the isolated components and the complex. Gibbs free energy (∆G) was calculated using the following equation:

(2) ∆G=∆H−T∆S

where T is the temperature in Kelvin (assumed to be 298.15 K in this study).

In addition, global reactivity descriptors such as hardness (η), electrophilicity (ω), chemical potential (µ), and electronegativity (χ) were computed to further assess the stability and reactivity of the ICs. These descriptors were derived using the following relations [36].

Hardness (η):

(3) $$\eta =\frac{I-A}{2}$$

where I is the ionization potential and A is the electron affinity.

Electrophilicity (ω):

(4) $$\omega =\frac{\mu^{2}}{2\eta }$$

where μ is the chemical potential.

Chemical potential (μ):

(5) $$\mu =\frac{I+A}{2}$$

Electronegativity (χ):

(6) $$\chi =\frac{I+A}{2}$$

The electronic properties of the ICs were derived from the calculated molecular orbital energies, offering detailed insights into the nature of the interactions between β-CD and PMT. These properties shed light on key factors such as the strength of the host–guest interactions, the stability of the complexes, and their electronic configurations. Additionally, they provide a quantitative basis for evaluating the complexes’ potential for further chemical reactivity and functional applications [37].

## Figures and Tables

**Table 1:** FMO energies and global reactivity descriptors of the β-CD, PMT, PMT:β-CD ICs Ori-1, and PMT:β-CD ICs Ori-2.

Compounds	*E*_HOMO_ (eV)	*E*_LUMO_ (eV)	∆E (eV)	μ (eV)	χ (eV)	ƞ (eV)	S (eV^−1^)	ω (eV)
β-CD	−6.549	−0.849	5.7	−3.699	3.699	2.85	1.425	2.400456
PMT	−5.976	−1.85	4.126	−3.913	3.913	2.063	1.0315	3.710996
Ori-1	−6.288	−1.951	4.337	−4.1195	4.1195	2.1685	1.08425	3.912908
Ori-2	−6.246	−2.177	4.069	−4.2115	4.2115	2.0345	1.01725	4.35899

**Table 2:** Hydrogen bond distances (Å) for PMT:β-CD ICs determined via the B3LYP-GD3/6-31G(d) method.

ICs	H-Bonding	Distance (Å)
Ori-1	(β-CD) C_1_-O-----H-C_18_(PMT)	2.915
	(β-CD) C_6_-O-----H-C_4_(PMT)	2.176
	(β-CD) C_6_-H-----N-C_10_(PMT)	2.393
Ori-2	(β-CD) C_6_-O-----H-C_21_(PMT)	2.616
	(β-CD) C_5_-O----HN-C_17_(PMT)	2.162
	(β-CD) C_2_-O----H-C_14_(PMT)	2.557
	(β-CD) C_3_-OH---O-C_15_(PMT)	1.796

## Data Availability

Data are available in the Supplementary documents.

## Supplementary Materials

The following supporting information can be downloaded at https://www.mdpi.com/article/10.3390/bioengineering13050583/s1: The optimized geometrical parameters for β-CD (Table S1), PMT (Table S2), PMT:β-CD ICs with Ori-1 (Table S3), and PMT:β-CD ICs with Ori-2 (Table S4) are provided.
