# Supplementary material for: Bioengineering Insights into Orientation and Structural Stability of Phenyl Methyl Thiazole Derivative with β-Cyclodextrin Through Computational Modeling
Source: Bioengineering (Basel). 2026 May 19;13(5):583. doi: 10.3390/bioengineering13050583 (PMC13203465; doi:10.3390/bioengineering13050583)
Supplement: Supplementary file 1 [file bioengineering-13-00583-s001.zip › bioengineering-4302933-supplementary.pdf]

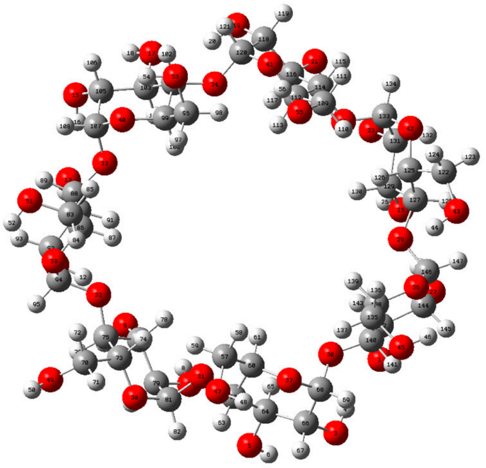

Table S1. Optimized geometrical parameters for β-CD.

Tag	Symbol	NA	NB	NC	Bond	Angle	Dihedral	X	Y	Z
1	O							4.507519	-4.05971	-1.90544
2	H	1			0.945566			4.902682	-3.81868	-2.72997
3	O	1	2		3.004973	136.3291		3.071613	-6.45771	-0.80197
4	H	3	1	2	0.950045	10.18715	-70.3487	3.386327	-5.67757	-1.24348
5	O	3	1	2	2.86551	121.9801	-84.1669	0.263299	-6.86239	-1.20291
6	H	5	3	1	0.944324	59.22113	85.68876	0.889883	-7.14916	-1.8486
7	O	5	3	1	3.286987	124.7752	1.812102	-1.68869	-4.86927	-2.94116
8	H	7	5	3	0.94425	152.7508	44.86298	-1.88871	-4.34011	-3.6972
9	O	7	5	3	2.847165	152.9523	-110.054	-3.7774	-2.95058	-3.19067
10	H	9	7	5	0.946334	98.47855	-104.113	-4.45521	-3.52401	-3.51826
11	O	9	7	5	2.98988	169.0883	-0.4038	-6.0079	-0.98279	-2.88731
12	H	11	9	7	0.949967	16.6218	88.78154	-5.14788	-1.38207	-2.94538
13	O	11	9	7	2.847049	124.1257	86.5802	-5.63204	1.78552	-2.33877
14	H	13	11	9	0.945512	54.64167	92.08981	-5.70593	1.403595	-3.20055
15	O	13	11	9	2.939007	171.2009	-107.056	-5.39385	4.545968	-1.35848
16	H	15	13	11	0.950756	8.874557	132.1989	-5.33471	3.671915	-1.7279
17	O	15	13	11	2.827829	120.4271	123.7022	-2.91748	5.904728	-1.49273
18	H	17	15	13	0.945821	53.37501	91.98272	-3.60288	5.915162	-2.14442
19	O	17	15	13	2.935113	168.8256	-5.01437	-0.14591	6.813931	-1.81923
20	H	19	17	15	0.948061	15.50026	4.942281	-0.94695	6.306833	-1.81846
21	O	19	17	15	2.853792	137.2485	-7.67109	2.386552	5.643372	-2.4197
22	H	21	19	17	0.944679	137.0124	24.998	2.703659	4.874591	-2.86785
23	O	21	19	17	3.023866	154.5125	9.474973	4.220482	3.400147	-3.28481
24	H	23	21	19	0.943359	88.80538	135.5093	4.680552	4.014366	-3.83344
25	O	23	21	19	2.782592	150.6994	-55.9463	5.191359	0.809141	-2.99002
26	H	25	23	21	0.944915	57.74318	111.5859	4.673149	1.071782	-3.73523
27	O	1	3	5	2.837211	165.9868	145.6618	6.234391	-1.85621	-2.36617
28	H	27	1	3	0.950431	112.8295	-128.464	5.764491	-1.05643	-2.57322
29	O	25	23	21	2.799148	114.5504	-31.0538	5.176045	-0.14628	-0.35902
30	O	1	27	29	2.779273	115.9192	-9.00411	2.905167	-3.8946	0.359417
31	O	7	5	3	2.77977	56.92056	-92.102	-1.30646	-4.60671	-0.20034
32	O	11	9	7	2.784811	59.10222	10.14934	-4.64721	-1.95642	-0.66116
33	O	13	11	9	2.790556	114.627	-63.7188	-4.44314	2.331611	0.126092
34	O	17	15	13	2.792373	116.2816	-66.4781	-0.86932	4.904504	0.120324
35	O	21	19	17	2.824558	116.2483	-60.6872	3.320956	3.689386	-0.60671
36	O	29	25	23	2.274919	152.0314	-173.267	5.689516	-1.70636	1.215073
37	O	30	1	27	2.281226	154.5038	-162.707	1.554793	-4.73952	1.992384
38	O	31	7	5	2.296404	89.46377	-110.386	-3.46883	-5.29801	0.145644
39	O	32	11	9	2.288981	90.50191	-174.606	-6.09305	-0.85435	0.72967

40	O	33	13	11	2.282514	152.3706	-165.564	-3.87129	3.741892	1.827255
41	O	34	17	15	2.298477	142.5567	-144.268	0.929057	5.551504	1.397124
42	O	35	21	19	2.299903	125.3675	-140.035	5.498223	3.459962	0.09787
43	O	36	29	25	2.877435	71.18188	118.4031	7.0005	0.763487	1.893891
44	H	43	36	29	0.944038	42.54905	109.7883	6.289157	0.239434	2.226407
45	O	36	29	25	2.794768	156.9391	-114.914	5.203906	-3.66698	3.146621
46	H	45	36	29	0.944461	58.88711	-171.507	6.072433	-3.32269	3.008329
47	O	37	30	1	2.750354	157.0137	-115.119	-0.7667	-5.10287	3.421754
48	H	47	37	30	0.944542	56.80329	-171.244	0.070253	-5.4408	3.700091
49	O	38	31	7	2.87838	150.5922	-104.372	-6.21071	-4.72114	0.80474
50	H	49	38	31	0.942026	133.9153	-125.083	-6.80981	-5.04953	1.453318
51	O	39	32	11	2.7902	156.0266	-108.588	-6.883	1.106923	2.55028
52	H	51	39	32	0.944337	58.82091	-174.084	-7.4591	0.395997	2.316901
53	O	40	33	13	2.79272	156.4781	-116.395	-2.15078	5.155418	3.512807
54	H	53	40	33	0.944309	59.44346	-172.486	-3.08036	5.141005	3.678331
55	O	41	34	17	2.740016	122.0787	156.0011	1.897711	4.001013	3.438049
56	H	55	41	34	0.944804	58.93246	-100.838	1.340588	4.74392	3.612282
57	C	47	37	30	1.395888	57.81939	-22.4959	-0.54322	-3.96602	2.643207
58	H	57	47	37	1.088316	110.9677	-90.9542	-0.07636	-3.17752	3.230351
59	H	57	47	37	1.079724	107.2713	151.1527	-1.50702	-3.61087	2.310386
60	C	37	30	1	1.416501	95.85105	-104.307	0.342842	-4.25346	1.44339
61	H	60	37	30	1.08491	109.7959	-30.7023	0.527284	-3.32865	0.906995
62	C	31	7	5	1.411877	121.6867	14.21135	-0.25535	-5.27062	0.46883
63	H	62	31	7	1.084493	110.3454	-104.622	-0.63897	-6.11832	1.025933
64	C	5	3	1	1.400638	56.94233	-60.6025	0.786919	-5.74865	-0.53419
65	H	64	5	3	1.08697	110.3942	90.85283	0.96776	-4.937	-1.23421
66	C	3	1	30	1.395114	113.1602	13.29535	2.099248	-6.12836	0.142684
67	H	66	3	1	1.084562	106.5316	-158.642	1.92785	-7.02161	0.733447
68	C	37	30	1	1.388263	35.1112	25.01194	2.568114	-5.0354	1.090768
69	H	68	37	30	1.081103	106.4353	119.7172	3.41149	-5.37127	1.677876
70	C	49	38	31	1.404247	54.50104	-41.0303	-5.08608	-4.15938	1.430478
71	H	70	49	38	1.085393	111.3098	-85.6383	-4.72128	-4.79527	2.230886
72	H	70	49	38	1.085699	110.5339	153.3704	-5.32662	-3.18372	1.84152
73	C	38	31	7	1.406842	95.01046	-103.331	-3.96714	-4.01096	0.41839
74	H	73	38	31	1.083814	110.0633	-29.839	-3.19177	-3.39881	0.864181
75	C	32	11	9	1.410007	107.372	-39.9589	-4.41198	-3.32952	-0.87884
76	H	75	32	11	1.084709	110.4661	-45.9935	-5.31087	-3.81522	-1.2431
77	C	9	7	5	1.415662	57.51473	4.000336	-3.32	-3.41761	-1.93498
78	H	77	9	7	1.081304	105.3622	88.9752	-2.51205	-2.75388	-1.65948
79	C	7	5	3	1.391261	98.38084	-136.099	-2.76881	-4.82875	-2.06521
80	H	79	7	5	1.092506	110.4549	-101.791	-3.56913	-5.48486	-2.41532
81	C	31	7	5	1.386404	59.44554	-92.5549	-2.35844	-5.36145	-0.69614
82	H	81	31	7	1.07706	111.0434	94.32174	-2.08827	-6.40163	-0.76741
83	C	51	39	32	1.398368	57.0356	-27.4112	-5.56141	0.665087	2.433587
84	H	83	51	39	1.087561	111.0675	-90.4371	-5.36823	-0.17087	3.101901
85	H	83	51	39	1.08085	107.2584	151.5408	-4.92189	1.485751	2.726432
86	C	39	32	11	1.41857	96.14883	-101.039	-5.22867	0.233267	1.016507
87	H	86	39	32	1.085082	109.8929	-30.5245	-4.19553	-0.09624	0.978507
88	C	33	13	11	1.406231	58.09281	-41.6125	-5.43123	1.341667	-0.01946
89	H	88	33	13	1.08395	110.6137	-87.7382	-6.42032	1.769839	0.095915
90	C	13	11	9	1.403492	57.29583	-66.0723	-5.29433	0.778091	-1.4218
91	H	90	13	11	1.087454	109.7905	91.1891	-4.25866	0.48005	-1.56711
92	C	11	9	7	1.396095	116.502	19.41694	-6.18932	-0.43806	-1.61474
93	H	92	11	9	1.084379	106.6613	-155.812	-7.22481	-0.12135	-1.55684
94	C	39	32	11	1.390069	34.64684	28.41207	-5.93881	-1.4605	-0.51173
95	H	94	39	32	1.080826	106.3556	120.3497	-6.66289	-2.26196	-0.55117
96	C	53	40	33	1.398633	56.71455	-26.5387	-1.79417	3.960812	2.878836
97	H	96	53	40	1.087615	111.0044	-89.7716	-2.04591	3.101566	3.496275
98	H	96	53	40	1.077933	107.3619	152.3004	-0.72469	3.974614	2.744779

99	C	40	33	13	1.420534	96.71821	-107.715	-2.4819	3.797877	1.536764
100	H	99	40	33	1.085439	109.7576	-30.2683	-2.16321	2.863033	1.086565
101	C	34	17	15	1.408737	58.08408	-37.8837	-2.20888	4.941358	0.554801
102	H	101	34	17	1.083597	110.3264	-89.0022	-2.4213	5.886603	1.040159
103	C	17	15	13	1.400981	58.21452	-66.3952	-3.10403	4.790785	-0.66382
104	H	103	17	15	1.087887	109.8682	92.45081	-2.81531	3.884518	-1.19186
105	C	15	13	11	1.393181	113.762	58.01529	-4.56412	4.677368	-0.24707
106	H	105	15	13	1.084885	106.4629	-158.083	-4.85321	5.599973	0.245072
107	C	40	33	13	1.388341	35.00313	21.19435	-4.73122	3.542631	0.755669
108	H	107	40	33	1.081386	106.474	119.99	-5.73245	3.530669	1.164064
109	C	55	41	34	1.395898	58.30541	115.4495	2.659887	4.277502	2.301751
110	H	109	55	41	1.079815	107.2006	-150.473	3.322704	3.438674	2.149957
111	H	109	55	41	1.088646	111.064	91.70721	3.269967	5.167392	2.446834
112	C	41	34	17	1.414652	97.55459	-143.019	1.783172	4.470239	1.076822
113	H	112	41	34	1.085317	109.854	-32.4841	1.195466	3.57325	0.909712
114	C	35	21	19	1.416547	57.70644	-34.5311	2.564889	4.811321	-0.18691
115	H	114	35	21	1.086522	109.5766	-88.6228	3.232935	5.640944	0.027501
116	C	21	19	17	1.396788	58.50306	-58.079	1.632479	5.227033	-1.32013
117	H	116	21	19	1.087262	110.4159	93.30628	1.009392	4.377074	-1.58747
118	C	19	17	15	1.384397	115.0489	-74.7996	0.735525	6.367418	-0.84956
119	H	118	19	17	1.085943	106.779	-138.13	1.373029	7.208617	-0.59413
120	C	41	34	17	1.390998	34.4556	-14.7304	0.010099	5.942196	0.428749
121	H	120	41	34	1.080885	106.042	120.0614	-0.52798	6.780406	0.848495
122	C	43	36	29	1.392303	126.5931	26.40158	6.587583	2.046039	1.54308
123	H	122	43	36	1.08213	106.4273	-130.258	7.45292	2.542034	1.123336
124	H	122	43	36	1.086972	111.7838	112.7201	6.272226	2.61975	2.410786
125	C	42	35	21	1.415186	99.418	-157.569	5.470436	2.102719	0.497708
126	H	125	42	35	1.085495	109.9163	-36.3698	4.508967	1.868228	0.943679
127	C	29	25	23	1.409746	57.72023	-45.9812	5.651013	1.138977	-0.69052
128	H	127	29	25	1.086051	110.5686	-85.3753	6.693701	1.094644	-0.99109
129	C	25	23	21	1.403355	58.34197	-34.9924	4.812954	1.584302	-1.88306
130	H	129	25	23	1.084774	110.099	90.98342	3.767778	1.413523	-1.64817
131	C	23	21	19	1.408431	106.9841	-113.718	5.009054	3.059776	-2.16857
132	H	131	23	21	1.086176	109.958	-117.882	6.057756	3.239768	-2.38676
133	C	35	21	19	1.38286	94.62158	-155.789	4.654248	3.869075	-0.92662
134	H	133	35	21	1.081612	110.6226	38.81902	4.87004	4.916981	-1.0854
135	C	45	36	29	1.397609	56.83039	-23.9871	4.270745	-2.68521	2.802177
136	H	135	45	36	1.08834	111.0469	-90.2574	4.386163	-1.80046	3.425375
137	H	135	45	36	1.080353	107.2854	151.9405	3.288966	-3.097	2.985754
138	C	36	29	25	1.420851	97.39156	-104.118	4.390715	-2.26717	1.347108
139	H	138	36	29	1.085593	109.8646	-29.2984	3.639789	-1.5134	1.131577
140	C	30	1	27	1.404143	58.44773	-37.9234	4.22844	-3.42495	0.359737
141	H	140	30	1	1.083963	110.4725	-88.0772	4.913671	-4.22234	0.623579
142	C	1	140	30	1.402298	37.42923	120.3716	4.54627	-2.95192	-1.04651
143	H	142	1	140	1.087922	109.8984	-118.552	3.79479	-2.22456	-1.34618
144	C	27	1	142	1.393539	57.73066	34.69966	5.921839	-2.30095	-1.08302
145	H	144	27	1	1.085049	106.361	88.90151	6.6583	-3.0579	-0.83405
146	C	36	29	25	1.392897	35.36544	23.21992	6.033495	-1.19868	-0.03556
147	H	146	36	29	1.077459	106.4418	119.6711	7.050256	-0.85145	0.045386

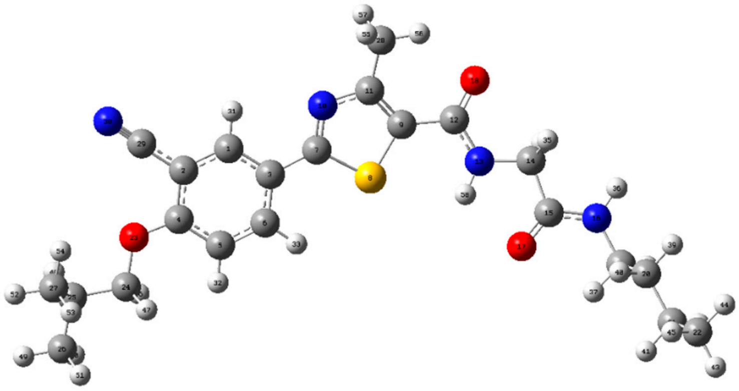

Table S2. Optimized geometrical parameters for PMT.

Tag	Symbol	NA	NB	NC	Bond	Angle	Dihedral	X	Y	Z
1	C							-3.50788	1.525323	0.312959
2	C	1			1.395483			-4.76402	0.917698	0.296166
3	C	1	2		1.403365	120.917		-2.35454	0.806685	-0.03747
4	C	2	1	3	1.418376	120.1885	-0.04464	-4.89261	-0.4441	-0.07901
5	C	4	2	1	1.400256	118.9065	0.213919	-3.74157	-1.16238	-0.42524
6	C	5	4	2	1.391801	120.1273	-0.15391	-2.49662	-0.54056	-0.40168
7	C	3	1	2	1.465977	118.9687	-179.972	-1.05652	1.48721	-0.00437
8	S	7	3	1	1.758873	122.7161	-175.623	0.434535	0.716601	-0.53027
9	C	8	7	3	1.754213	89.12625	179.7988	1.301627	2.191808	-0.14403
10	N	7	3	1	1.307783	123.6083	3.461079	-0.89609	2.720968	0.398615
11	C	10	7	3	1.373883	112.7667	-179.496	0.411016	3.137981	0.32693
12	C	9	8	7	1.486391	122.6701	179.2952	2.770793	2.329228	-0.32299
13	N	12	9	8	1.364852	116.1768	-14.4204	3.465294	1.169704	-0.51271
14	C	13	12	9	1.4421	120.5724	-177.644	4.901112	1.188107	-0.6459
15	C	14	13	12	1.532459	108.2142	170.989	5.403341	-0.25945	-0.6181
16	N	15	14	13	1.358477	114.8385	173.8271	6.730732	-0.40779	-0.86607
17	O	15	14	13	1.228555	121.4376	-5.74493	4.642245	-1.19758	-0.3945
18	O	12	9	8	1.232183	122.5345	164.8135	3.339729	3.422184	-0.31709
19	C	16	15	14	1.459887	122.8985	176.1428	7.409894	-1.69851	-0.80242
20	C	19	16	15	1.534193	113.5128	-96.6379	8.111456	-1.95557	0.537533
21	C	20	19	16	1.53403	112.477	-179.228	8.83266	-3.30906	0.571768
22	C	21	20	19	1.531981	112.9723	-179.818	9.533704	-3.57941	1.90684
23	O	4	2	1	1.347612	116.0096	-179.772	-6.14413	-0.9438	-0.07043
24	C	23	4	2	1.433948	119.825	-178.28	-6.36409	-2.32189	-0.40008
25	C	24	23	4	1.527779	108.1972	179.1132	-7.86077	-2.60668	-0.28622
26	C	25	24	23	1.535665	109.4244	175.2334	-8.14385	-4.04352	-0.74842
27	C	25	24	23	1.53447	111.6468	-60.4522	-8.3787	-2.35283	1.135719
28	C	11	10	7	1.499922	117.8824	-179.891	0.725783	4.537617	0.764772
29	C	2	1	3	1.432255	120.2203	179.746	-5.93065	1.663745	0.661868
30	N	29	2	1	1.163212	179.1334	-10.6087	-6.86783	2.282643	0.96474
31	H	1	2	4	1.08386	119.9626	-179.974	-3.41379	2.566057	0.600662
32	H	5	4	2	1.0831	120.4481	179.7417	-3.81041	-2.20468	-0.71159
33	H	6	5	4	1.086771	118.3723	179.6741	-1.6216	-1.12634	-0.6705
34	H	14	13	12	1.098246	110.9528	-67.7405	5.202578	1.680168	-1.58032
35	H	14	13	12	1.099567	110.8383	50.21362	5.36229	1.763111	0.170026
36	H	16	15	14	1.011421	118.3215	4.509604	7.293951	0.424821	-0.97795
37	H	19	16	15	1.092799	106.4774	24.88796	6.646648	-2.45958	-0.98251
38	H	19	16	15	1.097095	108.2066	140.4728	8.131672	-1.7471	-1.62722
39	H	20	19	16	1.099967	109.47	-57.1763	8.832699	-1.14876	0.734473
40	H	20	19	16	1.097788	108.6097	58.87381	7.362914	-1.91021	1.33926

41	H	21	20	19	1.098872	109.1856	-57.7279	8.107732	-4.10974	0.369515
42	H	21	20	19	1.099475	109.3657	58.00057	9.569006	-3.35054	-0.24366
43	H	22	21	20	1.095627	111.2921	179.8865	10.03733	-4.55241	1.901882
44	H	22	21	20	1.09686	111.2109	-60.0188	10.28941	-2.81366	2.120458
45	H	22	21	20	1.096596	111.1344	59.82163	8.81717	-3.57857	2.736961
46	H	24	23	4	1.098666	109.315	-60.1592	-6.00764	-2.51254	-1.42168
47	H	24	23	4	1.099862	109.382	58.40322	-5.79291	-2.95831	0.291592
48	H	25	24	23	1.098688	106.8402	57.93694	-8.36961	-1.9135	-0.9701
49	H	26	25	24	1.095062	110.4937	-177.78	-9.21791	-4.25365	-0.71119
50	H	26	25	24	1.09674	111.8412	-57.7684	-7.80526	-4.21534	-1.77734
51	H	26	25	24	1.09766	111.1894	62.94988	-7.64214	-4.77456	-0.10133
52	H	27	25	24	1.095662	110.5693	177.9335	-9.45915	-2.52692	1.18867
53	H	27	25	24	1.098207	110.892	-62.6949	-7.89635	-3.02849	1.85466
54	H	27	25	24	1.093499	111.2201	57.74937	-8.1835	-1.32389	1.450242
55	H	28	11	10	1.095863	109.7167	52.72827	0.327805	4.708412	1.771429
56	H	28	11	10	1.088582	111.1491	175.1492	1.798077	4.724016	0.743507
57	H	28	11	10	1.096918	109.6771	-64.5491	0.22997	5.253573	0.097835
58	H	13	12	9	1.015411	123.3683	-13.6044	3.073453	0.249193	-0.33899

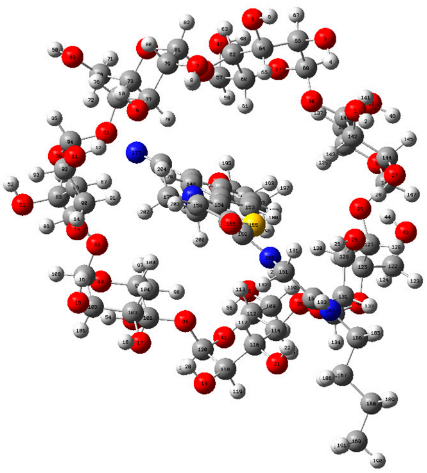

Table S3. Optimized geometrical parameters for PMT:β-CD ICs (Ori 1).

Tag	Symbol	NA	NB	NC	Bond	Angle	Dihedral	X	Y	Z
1	O							-1.85461	-0.08352	-0.43613
2	H	1			0.945382			-1.7279	-0.8471	-0.97893
3	O	1	2		3.030675	136.8213		-2.41734	0.480775	2.487888
4	H	3	1	2	0.949659	10.6164	-71.6818	-2.40734	0.247474	1.567387
5	O	3	1	2	2.875316	122.8832	-85.9464	-5.07425	0.523902	3.586269
6	H	5	3	1	0.943919	61.23448	84.12753	-4.63084	-0.30515	3.502295
7	O	5	3	1	3.156165	124.9642	5.126018	-7.69936	-0.22098	2.000294
8	H	7	5	3	0.944236	149.6422	48.42261	-8.16815	-0.81201	1.4324
9	O	7	5	3	2.830487	156.855	-108.53	-10.1635	0.236251	0.684726
10	H	9	7	5	0.946416	98.80594	-103.757	-10.7484	-0.07552	1.36026
11	O	9	7	5	2.92771	171.4436	7.653851	-12.6214	1.133797	-0.62853
12	H	11	9	7	0.949868	16.1058	78.01677	-11.7191	0.905582	-0.4388
13	O	11	9	7	2.829363	123.9578	75.73458	-12.7207	2.164164	-3.26174
14	H	13	11	9	0.945634	52.19312	97.55152	-12.9063	1.281662	-2.9772
15	O	13	11	9	2.9346	172.9871	-88.6753	-12.7621	3.554445	-5.84578
16	H	15	13	11	0.950668	9.909149	101.0837	-12.5962	3.060079	-5.05089
17	O	15	13	11	2.809933	124.6246	100.1112	-10.613	3.626683	-7.65454
18	H	17	15	13	0.945849	52.2288	92.7415	-11.3318	3.012296	-7.63316
19	O	17	15	13	2.943696	175.5665	-98.8642	-8.31022	3.908191	-9.46658
20	H	19	17	15	0.948476	15.4207	113.0762	-8.90748	3.633443	-8.78291

21	O	19	17	15	2.853471	128.8635	100.1764	-5.6924	2.799667	-9.22069
22	H	21	19	17	0.945567	129.706	26.18987	-5.40663	2.019816	-8.76873
23	O	21	19	17	2.905217	138.6277	2.669417	-4.22777	1.044048	-7.42822
24	H	23	21	19	0.948664	114.8688	104.0583	-4.09311	0.163382	-7.75419
25	O	23	21	19	2.825961	158.1681	-90.3137	-2.60227	0.290293	-5.24288
26	H	25	23	21	0.944414	61.7171	130.5736	-3.41729	-0.1686	-5.3736
27	O	1	3	5	2.845263	164.9081	146.0995	-0.76964	-0.12823	-3.06603
28	H	27	1	3	0.950527	108.8634	-130.743	-1.47469	-0.00492	-3.69148
29	O	25	23	21	2.806335	112.7184	-4.88065	-1.59283	2.495841	-3.83147
30	O	1	27	29	2.778078	115.9252	-8.94597	-2.81773	2.370981	0.438835
31	O	7	5	3	2.782724	58.73564	-92.0565	-6.78441	2.403965	2.127027
32	O	11	9	7	2.830869	59.76183	0.500649	-10.7508	3.024546	0.34084
33	O	13	11	9	2.799411	114.8625	-65.6616	-11.387	4.598624	-3.62412
34	O	17	15	13	2.798861	117.1562	-68.5842	-8.34471	5.075181	-6.88611
35	O	23	21	19	2.780048	61.39297	-91.1675	-4.1656	3.782665	-6.95411
36	O	29	25	23	2.269782	150.1559	177.5323	-0.39002	3.344896	-2.10396
37	O	30	1	27	2.285378	155.1541	-161.664	-3.49691	3.895003	2.000572
38	O	31	7	5	2.295001	89.49491	-114.565	-8.66012	2.978819	3.317939
39	O	32	11	9	2.296506	89.11137	-171.241	-12.2692	4.69324	-0.08773
40	O	33	13	11	2.288773	152.1321	-162.222	-10.9618	6.517815	-4.79646
41	O	34	17	15	2.296816	147.7719	-145.619	-6.58584	6.410328	-7.51787
42	O	35	23	21	2.296943	89.70121	-119.183	-1.88536	3.766532	-7.23015
43	O	36	29	25	2.972087	69.09869	126.7128	0.548611	4.454971	-4.69626
44	H	43	36	29	0.943991	46.39059	106.1673	0.04486	4.808841	-3.98063
45	O	36	29	25	2.791803	157.1523	-106.325	0.055015	4.604979	0.347219
46	H	45	36	29	0.944646	57.85355	-171.89	0.760369	4.392684	-0.24419
47	O	37	30	1	2.721911	159.6699	-109.627	-5.14831	5.503715	3.447538
48	H	47	37	30	0.944285	55.9429	-175.125	-4.20837	5.49302	3.537401
49	O	38	31	7	2.879636	151.5299	-105.424	-11.2443	4.233142	3.520585
50	H	49	38	31	0.942153	140.2744	-115.367	-11.6721	4.942966	3.968684
51	O	39	32	11	2.793138	156.5231	-103.594	-13.3891	6.843958	-1.47409
52	H	51	39	32	0.944075	59.82142	-179.105	-13.793	6.528348	-0.68129
53	O	40	33	13	2.795666	156.9817	-124.725	-9.4203	8.423564	-6.14096
54	H	53	40	33	0.944493	59.15941	-168.845	-10.3201	8.600529	-5.91498
55	O	41	34	17	2.741585	123.1946	168.6849	-5.0053	7.757255	-5.72791
56	H	55	41	34	0.944919	57.92611	-100.147	-5.70343	8.183656	-6.20084
57	C	47	37	30	1.396534	58.86532	-26.9475	-5.46663	5.131558	2.139685
58	H	57	47	37	1.087557	110.9148	-91.8866	-5.09491	5.86353	1.426366
59	H	57	47	37	1.080057	107.2507	150.273	-6.54395	5.108581	2.066423
60	C	37	30	1	1.418724	97.41891	-103.494	-4.89343	3.768078	1.785174
61	H	60	37	30	1.084557	109.6734	-32.9643	-5.0834	3.551443	0.739591
62	C	31	7	5	1.413124	118.4555	11.56517	-5.48665	2.640954	2.633565
63	H	62	31	7	1.084111	110.2678	-100.138	-5.54023	2.966308	3.666314
64	C	5	3	1	1.400206	56.64589	-59.2796	-4.65554	1.368154	2.550655
65	H	64	5	3	1.087075	110.5633	90.73728	-4.84742	0.910286	1.583562
66	C	3	1	30	1.395909	113.1442	13.34555	-3.16276	1.643791	2.688657
67	H	66	3	1	1.084465	106.4709	-158.044	-2.97297	1.958347	3.708998
68	C	37	30	1	1.386296	35.02663	24.85265	-2.71385	2.773679	1.774129
69	H	68	37	30	1.081261	106.2671	119.5386	-1.69703	3.063648	2.000226
70	C	49	38	31	1.405532	54.77476	-37.3209	-10.158	4.729681	2.779707
71	H	70	49	38	1.085365	111.2539	-85.9826	-9.50039	5.330332	3.40004
72	H	70	49	38	1.084088	110.8146	152.9742	-10.5027	5.336294	1.94999
73	C	38	31	7	1.409697	96.20077	-101.517	-9.35113	3.566951	2.23912
74	H	73	38	31	1.084294	109.863	-28.0944	-8.64286	3.959789	1.518206
75	C	32	11	9	1.41141	107.4458	-36.8022	-10.2165	2.513173	1.542971
76	H	75	32	11	1.084471	110.4132	-51.4187	-11.014	2.220981	2.217237
77	C	9	7	5	1.417908	57.9997	4.262072	-9.37063	1.299593	1.185884
78	H	77	9	7	1.081039	105.1305	88.97465	-8.7011	1.557715	0.377334
79	C	7	5	3	1.391286	101.6068	-134.805	-8.55397	0.81178	2.37274

80	H	79	7	5	1.092831	110.4006	-100.895	-9.24606	0.472822	3.14759
81	C	31	7	5	1.386891	59.54676	-96.6064	-7.76556	1.958621	3.000229
82	H	81	31	7	1.077765	110.8056	94.86957	-7.33069	1.634545	3.931596
83	C	51	39	32	1.401169	56.87889	-34.5842	-12.0076	6.628688	-1.3814
84	H	83	51	39	1.085061	111.3563	-90.2279	-11.5868	7.129722	-0.5158
85	H	83	51	39	1.080778	107.2846	151.7423	-11.5563	7.053953	-2.26655
86	C	39	32	11	1.419613	96.54359	-101.892	-11.6716	5.150509	-1.29151
87	H	86	39	32	1.080777	109.6501	-33.431	-10.5984	5.038501	-1.2299
88	C	33	13	11	1.410738	57.87631	-41.8161	-12.1825	4.340971	-2.48794
89	H	88	33	13	1.083747	110.3348	-86.8282	-13.2174	4.593148	-2.68775
90	C	13	11	9	1.40688	57.92435	-67.5845	-12.0831	2.852737	-2.21359
91	H	90	13	11	1.085038	109.4066	91.9311	-11.034	2.577019	-2.18674
92	C	11	9	7	1.395903	116.1652	7.808319	-12.7212	2.503632	-0.87781
93	H	92	11	9	1.084744	106.5375	-154.55	-13.7804	2.733327	-0.92058
94	C	39	32	11	1.387577	34.50805	27.75305	-12.1025	3.352122	0.226882
95	H	94	39	32	1.080838	106.4166	120.266	-12.6091	3.193997	1.168482
96	C	53	40	33	1.398466	56.7171	-22.5398	-8.89312	7.538706	-5.19502
97	H	96	53	40	1.087686	111.1121	-90.1558	-8.93324	7.964661	-4.19501
98	H	96	53	40	1.07919	107.3086	151.6165	-7.85802	7.373468	-5.45177
99	C	40	33	13	1.419585	97.56058	-112.717	-9.62858	6.212777	-5.17691
100	H	99	40	33	1.085544	109.8629	-29.0695	-9.17066	5.569052	-4.43237
101	C	34	17	15	1.408453	58.28136	-34.6973	-9.64282	5.485808	-6.52549
102	H	101	34	17	1.0837	110.1442	-89.9924	-10.0496	6.145339	-7.28309
103	C	17	15	13	1.400661	58.87278	-66.7009	-10.5304	4.256882	-6.40639
104	H	103	17	15	1.08823	109.8302	92.78506	-10.0891	3.578539	-5.67883
105	C	15	13	11	1.393257	115.1439	32.28464	-11.9227	4.662843	-5.93518
106	H	105	15	13	1.085064	106.4292	-153.942	-12.3552	5.315572	-6.68634
107	C	40	33	13	1.390057	34.64502	15.32072	-11.8337	5.447872	-4.63166
108	H	107	40	33	1.081539	106.3051	120.2398	-12.793	5.875042	-4.37282
109	C	55	41	34	1.399375	58.15737	114.3975	-4.50094	6.725818	-6.52792
110	H	109	55	41	1.080392	107.2386	-150.603	-3.69368	6.261418	-5.98029
111	H	109	55	41	1.087985	110.6753	91.65078	-4.09605	7.122824	-7.45644
112	C	41	34	17	1.411842	97.33599	-130.537	-5.56772	5.694736	-6.85106
113	H	112	41	34	1.084466	109.6466	-33.7473	-5.95894	5.277157	-5.92984
114	C	35	23	21	1.416196	115.9277	8.780466	-5.04733	4.566831	-7.73721
115	H	114	35	23	1.087367	109.5718	-98.0971	-4.50599	4.99355	-8.57817
116	C	21	19	17	1.396708	58.7	-62.895	-6.18735	3.707019	-8.28126
117	H	116	21	19	1.086666	110.6058	93.46352	-6.64713	3.182312	-7.44812
118	C	19	17	15	1.384156	115.1303	34.0872	-7.23242	4.601896	-8.94407
119	H	118	19	17	1.085517	106.4032	-148.526	-6.75768	5.105737	-9.78019
120	C	41	34	17	1.391653	34.63246	-2.35414	-7.68529	5.680094	-7.95908
121	H	120	41	34	1.080434	105.9742	119.905	-8.33541	6.389304	-8.45072
122	C	43	36	29	1.393989	127.2473	20.46838	-0.15362	4.497368	-5.89971
123	H	122	43	36	1.082203	106.5445	-123.936	0.462685	3.99521	-6.63398
124	H	122	43	36	1.087139	111.653	119.0624	-0.30129	5.518521	-6.24221
125	C	42	35	23	1.410609	98.34614	-101.349	-1.51417	3.800302	-5.86968
126	H	125	42	35	1.086435	109.881	-34.1737	-2.23017	4.391804	-5.30593
127	C	29	25	23	1.412101	57.70436	-49.2061	-1.50624	2.395916	-5.23737
128	H	127	29	25	1.086006	110.7738	-86.301	-0.61774	1.844425	-5.53033
129	C	25	23	21	1.405876	57.14091	-12.001	-2.74854	1.624813	-5.6602
130	H	129	25	23	1.080571	110.0098	88.92316	-3.59023	2.079267	-5.15754
131	C	23	21	19	1.402965	105.9091	-131.411	-3.01873	1.699493	-7.15084
132	H	131	23	21	1.086155	110.616	-104.785	-2.20016	1.239025	-7.69641
133	C	35	23	21	1.387362	59.95775	-101.073	-3.09155	3.160503	-7.57388
134	H	133	35	23	1.078767	110.7599	95.71723	-3.16952	3.241983	-8.64673
135	C	45	36	29	1.396706	56.87091	-22.3611	-1.11642	4.771262	-0.39499
136	H	135	45	36	1.089043	110.9275	-90.0794	-1.02488	5.610002	-1.08357
137	H	135	45	36	1.080727	107.3953	152.0241	-1.91151	4.994907	0.301998
138	C	36	29	25	1.420716	97.56369	-94.2271	-1.46899	3.531563	-1.19875

139	H	138	36	29	1.084217	109.8006	-29.1744	-2.38328	3.718578	-1.75067
140	C	30	1	27	1.404065	58.43269	-37.9028	-1.65848	2.274205	-0.34741
141	H	140	30	1	1.084242	110.408	-87.6917	-0.7867	2.128902	0.280659
142	C	1	140	30	1.402149	37.54432	120.3219	-1.81501	1.060048	-1.24651
143	H	142	1	140	1.087891	109.8298	-118.44	-2.74801	1.154007	-1.79805
144	C	27	1	142	1.393707	57.51335	34.67229	-0.65191	0.977932	-2.2264
145	H	144	27	1	1.084877	106.2338	88.84464	0.257105	0.828408	-1.65344
146	C	36	29	25	1.392129	35.6217	32.76294	-0.49124	2.282944	-2.99841
147	H	146	36	29	1.077322	106.4568	119.2238	0.421067	2.278501	-3.57139
148	C	32	11	9	4.070939	121.1622	83.59898	-7.50983	4.106742	-1.87225
149	C	148	32	11	1.385085	109.7606	152.6041	-7.11879	5.391393	-1.53281
150	C	148	32	11	1.389468	128.5576	-40.1315	-6.69026	3.304996	-2.65719
151	C	149	148	32	1.402813	121.0142	168.7858	-5.89527	5.915355	-1.97593
152	C	151	149	148	1.390477	118.4368	-1.27972	-5.06722	5.104623	-2.74434
153	C	152	151	149	1.382035	119.9125	0.725981	-5.46533	3.820858	-3.06601
154	C	150	148	32	1.47413	119.8058	16.31128	-7.14381	1.959538	-3.05354
155	S	154	150	148	1.731754	122.2407	175.9275	-6.22059	0.948497	-4.11394
156	C	155	154	150	1.743123	89.20551	-178.852	-7.46991	-0.26244	-4.00759
157	N	154	150	148	1.282046	123.7886	-3.0306	-8.25058	1.438452	-2.66993
158	C	156	155	154	1.354089	109.3058	-0.40881	-8.45519	0.181317	-3.19159
159	C	156	155	154	1.482702	122.4834	-179.712	-7.38332	-1.56049	-4.71891
160	N	159	156	155	1.358696	116.1763	-18.2562	-6.49651	-1.62292	-5.74639
161	C	160	159	156	1.438131	118.7171	-178.459	-6.39213	-2.84594	-6.49574
162	C	161	160	159	1.517557	110.0774	177.7897	-5.38977	-2.68212	-7.62331
163	N	162	161	160	1.336662	114.9824	173.1186	-5.10799	-3.80264	-8.29539
164	O	162	161	160	1.210707	121.6634	-7.49377	-4.90076	-1.60658	-7.88767
165	O	159	156	155	1.20286	122.6877	159.7358	-8.02687	-2.52342	-4.39412
166	C	163	162	161	1.45598	123.1209	175.73	-4.24144	-3.83219	-9.46505
167	C	166	163	162	1.5269	113.1888	-86.8356	-4.98282	-3.55051	-10.7698
168	C	167	166	163	1.529177	112.3533	-179.42	-4.05798	-3.60019	-11.9866
169	C	168	167	166	1.527625	112.7785	-179.871	-4.7894	-3.31655	-13.2974
170	O	151	149	148	1.323734	116.4483	179.8639	-5.61335	7.153034	-1.6005
171	C	170	151	149	1.423404	121.2611	-169.927	-4.51132	7.861018	-2.15759
172	C	171	170	151	1.522354	108.1961	171.1977	-4.5902	9.307595	-1.68988
173	C	172	171	170	1.531278	109.1592	174.6001	-3.34292	10.05813	-2.16507
174	C	172	171	170	1.530102	111.5487	-61.8049	-5.87155	9.986869	-2.17766
175	C	158	156	155	1.497564	128.0789	178.871	-9.73267	-0.50931	-2.82584
176	C	149	148	32	1.441351	119.989	-11.4705	-7.97645	6.213606	-0.7168
177	N	176	149	148	1.13675	178.5602	3.288437	-8.67101	6.840129	-0.07085
178	H	148	32	11	1.072613	14.40744	15.65656	-8.44984	3.724483	-1.52477
179	H	152	151	149	1.070692	120.5079	-179.377	-4.12163	5.467399	-3.09169
180	H	153	152	151	1.074535	118.1637	-178.488	-4.79298	3.21023	-3.64021
181	H	161	160	159	1.083927	110.1703	-61.2176	-6.08054	-3.65639	-5.84691
182	H	161	160	159	1.08702	111.4907	57.08987	-7.34987	-3.13571	-6.92047
183	H	163	162	161	0.994175	118.2752	3.290944	-5.59735	-4.63323	-8.05242
184	H	166	163	162	1.081136	107.6395	35.49294	-3.45816	-3.10308	-9.31099
185	H	166	163	162	1.084603	107.352	150.9574	-3.77455	-4.81063	-9.49709
186	H	167	166	163	1.088733	109.2093	-57.51	-5.78535	-4.2764	-10.8897
187	H	167	166	163	1.086559	108.768	58.74832	-5.45075	-2.57224	-10.7019
188	H	168	167	166	1.08817	109.3038	-58.0221	-3.2552	-2.87691	-11.8581
189	H	168	167	166	1.088679	109.4904	58.0987	-3.58312	-4.57827	-12.0423
190	H	169	168	167	1.085126	111.0699	179.8857	-4.10801	-3.35678	-14.141
191	H	169	168	167	1.086306	111.1641	-60.1356	-5.57684	-4.04411	-13.4725
192	H	169	168	167	1.085922	111.1262	59.88441	-5.24547	-2.33111	-13.2858
193	H	171	170	151	1.086493	108.7765	-68.9539	-3.58954	7.402217	-1.81076
194	H	171	170	151	1.08317	109.2433	50.47862	-4.55907	7.802826	-3.23814
195	H	172	171	170	1.08754	107.3651	57.00006	-4.59655	9.297233	-0.60241
196	H	173	172	171	1.084599	110.5147	-178.637	-3.36502	11.08707	-1.82279
197	H	173	172	171	1.086577	111.7002	-58.8768	-2.4318	9.604733	-1.78435

198	H	173	172	171	1.086901	111.0811	61.84708	-3.28449	10.07155	-3.25032
199	H	174	172	171	1.085446	110.4568	179.6845	-5.91223	11.01433	-1.82999
200	H	174	172	171	1.087163	110.7867	-60.8762	-5.91131	10.00087	-3.264
201	H	174	172	171	1.083008	111.3145	59.7758	-6.75264	9.47517	-1.81057
202	H	175	158	156	1.085316	108.7167	-116.868	-10.5657	0.083144	-3.19058
203	H	175	158	156	1.076806	111.6533	4.4045	-9.78118	-1.50139	-3.24171
204	H	175	158	156	1.083965	109.1073	125.6994	-9.81013	-0.56356	-1.74601
205	H	160	159	156	0.995848	121.455	-20.7281	-6.16854	-0.79957	-6.20052

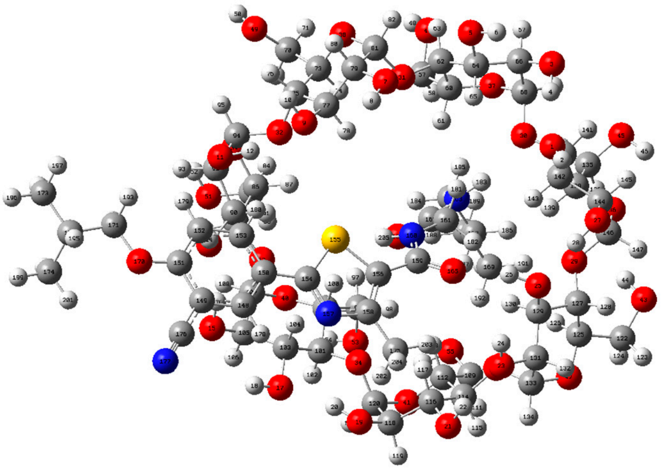

Table S4. Optimized geometrical parameters for PMT:β-CD ICs (Ori 2).

Tag	Symbol	NA	NB	NC	Bond	Angle	Dihedral	X	Y	Z
1	O							-4.545	2.429426	-3.54971
2	H	1			0.975653			-4.57029	1.940574	-4.39368
3	O	1	2		2.797809	135.1197		-4.89417	5.100724	-2.79474
4	H	3	1	2	0.982016	6.033961	-51.4877	-4.70364	4.197452	-3.12963
5	O	3	1	2	2.841545	104.3307	-96.0541	-2.255	6.146037	-2.66631
6	H	5	3	1	0.972969	49.957	88.87381	-2.87416	6.099688	-3.41542
7	O	5	3	1	3.386137	131.1478	-6.91889	0.758833	4.627915	-2.94568
8	H	7	5	3	0.971893	153.478	41.20468	1.38022	3.994971	-3.34296
9	O	7	5	3	2.892502	152.6418	-98.3743	3.151182	3.442497	-1.83302
10	H	9	7	5	0.974065	95.95982	-109.647	3.754568	4.156496	-2.10676
11	O	9	7	5	3.009151	166.7171	-13.6997	5.419676	2.511858	-0.08864
12	H	11	9	7	0.980671	13.40606	73.80807	4.562041	2.656862	-0.54158
13	O	11	9	7	2.974809	122.9399	96.78771	5.479323	-0.17247	1.192101
14	H	13	11	9	0.972464	60.65771	83.70123	5.778923	-0.07676	0.271903
15	O	13	11	9	2.967127	169.2125	-46.0652	5.01083	-2.73671	2.609499
16	H	15	13	11	0.980923	11.16565	55.48637	5.012736	-1.93918	2.038415
17	O	15	13	11	2.883706	124.3798	63.44886	3.08725	-4.72258	1.789778
18	H	17	15	13	0.973562	52.25656	91.3047	4.025299	-4.6613	1.536537
19	O	17	15	13	2.952274	156.478	6.627198	1.066002	-5.97252	0.038175
20	H	19	17	15	0.977498	7.731646	-25.1832	1.700459	-5.44464	0.561913
21	O	19	17	15	2.866851	157.207	-66.7972	-1.43762	-6.24762	-1.33114
22	H	21	19	17	0.974848	124.3542	71.09377	-1.71357	-5.69934	-2.08849
23	O	21	19	17	2.782508	129.5017	54.47509	-2.71591	-4.47689	-3.05533
24	H	23	21	19	0.972719	140.4359	33.57667	-2.5103	-3.80755	-3.73053
25	O	23	21	19	2.799363	150.3051	-60.3059	-3.88312	-1.98129	-3.55138
26	H	25	23	21	0.983715	86.38985	62.84957	-2.99408	-1.59962	-3.37353
27	O	1	3	5	2.799258	148.5314	-172.479	-5.69306	0.003725	-4.34581
28	H	27	1	3	0.986545	104.1027	-154.281	-5.03791	-0.67698	-4.06174
29	O	25	23	21	2.882254	115.4981	-48.6118	-5.79514	-1.09312	-1.586

30	O	1	3	5	2.882528	63.37147	89.87526	-4.55144	2.982181	-0.72069
31	O	7	5	3	2.817198	56.21907	-88.6386	-0.62935	4.917958	-0.51146
32	O	11	9	7	2.76469	59.34453	19.82798	3.031162	3.342324	1.028871
33	O	13	11	9	2.79882	110.9408	-56.1372	3.201455	-0.58055	2.766326
34	O	17	15	13	2.791751	116.4682	-64.1846	0.402378	-3.96167	1.869511
35	O	23	21	19	2.902651	63.33711	-94.3278	-3.4832	-4.35438	-0.25862
36	O	29	25	23	2.293789	144.0447	-174.578	-7.31544	0.549609	-1.08442
37	O	30	1	3	2.311473	140.4495	-27.5882	-4.06102	4.684766	0.763767
38	O	31	7	5	2.340101	89.38085	-113.541	1.053758	6.355571	0.24782
39	O	32	11	9	2.321259	92.08456	-177.898	3.832254	3.026687	3.184531
40	O	33	13	11	2.32154	154.1109	-167.684	1.934316	-1.7029	4.355111
41	O	34	17	15	2.337971	128.1481	-137.533	-1.42787	-5.201	2.631319
42	O	35	23	21	2.329009	87.28666	-123.588	-5.67289	-4.73038	-0.95732
43	O	29	25	23	2.956439	130.3445	-61.2414	-8.3569	-2.29427	-0.72859
44	H	43	29	25	0.97093	62.66363	163.6374	-7.96496	-1.61997	-0.15031
45	O	36	29	25	2.835854	153.3921	-93.8933	-8.10606	2.987901	0.128725
46	H	45	36	29	0.971763	55.60698	177.3969	-8.683	2.43999	-0.42918
47	O	37	30	1	2.846012	157.4865	-64.9939	-2.45303	6.56828	2.166096
48	H	47	37	30	0.968171	141.5005	-129.372	-2.25869	6.880453	3.061716
49	O	38	31	7	2.936186	147.22	-103.431	3.41937	6.704401	1.951752
50	H	49	38	31	0.968531	139.1204	-118.558	3.705818	7.239735	2.706349
51	O	39	32	11	2.823526	152.7497	-108.282	3.932659	1.652377	5.648976
52	H	51	39	32	0.971479	54.67425	179.9976	4.455385	2.43944	5.423009
53	O	40	33	13	2.976617	148.7111	-123.943	-0.5979	-3.09532	5.068766
54	H	53	40	33	0.97024	62.28845	-175.239	0.05647	-2.96026	5.772277
55	O	41	34	17	2.849181	111.3695	145.552	-3.37259	-3.52663	3.869214
56	H	55	41	34	0.975592	56.27298	-80.6943	-2.56493	-3.84132	4.316922
57	C	47	37	30	1.418826	55.7096	-53.7785	-2.19095	5.174807	2.114933
58	H	57	47	37	1.099889	111.4359	-85.1583	-2.75475	4.631139	2.887143
59	H	57	47	37	1.0992	111.7258	153.7941	-1.12278	4.956289	2.254547
60	C	37	30	1	1.440542	100.6335	-75.5302	-2.62125	4.638167	0.755465
61	H	60	37	30	1.096742	109.3161	-37.1671	-2.29247	3.594157	0.686287
62	C	31	7	5	1.439216	123.6579	8.18463	-1.97903	5.408552	-0.41644
63	H	62	31	7	1.094481	110.2129	-96.2806	-1.97709	6.479809	-0.19218
64	C	5	3	1	1.419936	58.77427	-68.1386	-2.7079	5.174118	-1.73547
65	H	64	5	3	1.101049	110.4928	92.4407	-2.46376	4.159888	-2.08767
66	C	3	1	27	1.416869	107.6593	126.8839	-4.22395	5.289763	-1.56081
67	H	66	3	1	1.096506	105.5502	-175.189	-4.45525	6.317051	-1.25502
68	C	37	30	1	1.395784	36.32936	47.43574	-4.72757	4.387477	-0.426
69	H	68	37	30	1.094419	106.0952	116.6587	-5.78885	4.573191	-0.23378
70	C	49	38	31	1.425937	53.76086	-43.6489	2.209656	6.04221	2.314232
71	H	70	49	38	1.098914	111.4252	-85.1616	1.457813	6.75349	2.683589
72	H	70	49	38	1.099172	110.9453	153.407	2.393223	5.285323	3.089864
73	C	38	31	7	1.430975	92.7878	-103.773	1.629332	5.350901	1.088678
74	H	73	38	31	1.097269	109.7386	-29.8434	0.847875	4.659415	1.428047
75	C	32	11	9	1.429813	105.1548	-43.6271	2.675447	4.527536	0.312558
76	H	75	32	11	1.096819	110.4778	-41.137	3.560526	5.152053	0.140435
77	C	9	7	5	1.444241	56.92295	-2.74341	2.116304	4.039152	-1.02131
78	H	77	9	7	1.092989	104.5647	88.27207	1.411564	3.225313	-0.83255
79	C	7	5	3	1.410519	99.76174	-129.881	1.392127	5.13978	-1.79394
80	H	79	7	5	1.106079	110.8578	-104.82	2.128091	5.920331	-2.06321
81	C	31	7	5	1.40476	59.26534	-96.034	0.365862	5.836619	-0.88424
82	H	81	31	7	1.091307	111.0164	94.16761	-0.06834	6.699237	-1.39248
83	C	51	39	32	1.419986	56.79568	-29.2699	2.772492	1.699447	4.831567
84	H	83	51	39	1.10104	111.2966	-90.2333	2.135668	2.559573	5.090269
85	H	83	51	39	1.093035	107.0112	151.6945	2.207076	0.78442	5.025874
86	C	39	32	11	1.446232	92.92593	-102.345	3.115443	1.781284	3.348053
87	H	86	39	32	1.096404	109.7231	-30.5683	2.190241	1.806952	2.760309
88	C	33	13	11	1.425671	57.97484	-48.619	3.982642	0.608306	2.860731

89	H	88	33	13	1.096512	110.7072	-85.5053	4.822028	0.466815	3.551926
90	C	13	11	9	1.427016	54.44843	-63.2686	4.518581	0.850003	1.452664
91	H	90	13	11	1.099794	110.1268	89.28052	3.66911	0.756391	0.760434
92	C	11	9	7	1.424233	117.3226	32.87344	5.125998	2.246702	1.279525
93	H	92	11	9	1.0951	106.4925	-159.652	6.079915	2.287542	1.815826
94	C	39	32	11	1.411007	34.89757	27.77813	4.184999	3.320389	1.850271
95	H	94	39	32	1.093745	106.1444	119.9087	4.661754	4.304533	1.871327
96	C	53	40	33	1.424593	52.23426	-28.2156	-0.41121	-2.06456	4.103292
97	H	96	53	40	1.09946	111.1595	-88.3971	-0.51737	-1.06975	4.559252
98	H	96	53	40	1.088408	106.2121	154.6864	-1.21102	-2.1823	3.374545
99	C	40	33	13	1.451663	93.47512	-113.86	0.93049	-2.116	3.391261
100	H	99	40	33	1.094956	109.3613	-27.5494	0.894976	-1.39152	2.571019
101	C	34	17	15	1.427726	59.05776	-36.1816	1.324927	-3.50343	2.858103
102	H	101	34	17	1.096323	109.8717	-90.2814	1.346163	-4.20392	3.701181
103	C	17	15	13	1.425788	57.38743	-64.4834	2.70806	-3.418	2.22241
104	H	103	17	15	1.100349	109.8348	91.40683	2.647131	-2.74077	1.357302
105	C	15	13	11	1.415928	114.9298	-2.73838	3.735708	-2.86111	3.212361
106	H	105	15	13	1.096348	106.1611	-155.254	3.853156	-3.58382	4.028366
107	C	40	33	13	1.411698	34.95429	15.06933	3.226177	-1.54029	3.809633
108	H	107	40	33	1.094355	106.256	119.9266	3.86494	-1.20079	4.630811
109	C	55	41	34	1.420313	55.2297	122.0872	-3.62292	-4.37297	2.756416
110	H	109	55	41	1.092851	106.8158	-153.113	-4.45202	-3.92776	2.200797
111	H	109	55	41	1.10149	111.4634	88.77776	-3.92456	-5.38248	3.077641
112	C	41	34	17	1.435423	95.11264	-156.244	-2.39979	-4.48357	1.856002
113	H	112	41	34	1.097886	109.6132	-25.8482	-2.03007	-3.47114	1.647098
114	C	35	23	21	1.438319	109.6614	7.550892	-2.61533	-5.19778	0.518704
115	H	114	35	23	1.09873	110.0181	-96.458	-3.08629	-6.17771	0.677245
116	C	21	19	17	1.416563	57.78627	-18.784	-1.27572	-5.3846	-0.21955
117	H	116	21	19	1.098469	111.0362	88.26963	-0.93967	-4.38882	-0.53912
118	C	19	17	15	1.414856	109.5948	-114.406	-0.1897	-5.97626	0.690101
119	H	118	19	17	1.100302	108.5974	-113.671	-0.47376	-7.01862	0.898561
120	C	41	34	17	1.40955	34.28476	-29.1213	-0.14548	-5.25584	2.048836
121	H	120	41	34	1.094611	105.3024	119.1486	0.468256	-5.82463	2.75451
122	C	43	29	25	1.417784	85.92584	48.98555	-7.63192	-3.50672	-0.60833
123	H	122	43	29	1.094313	105.9068	-112.251	-8.01876	-4.16639	-1.39108
124	H	122	43	29	1.099832	111.8555	131.0235	-7.81531	-3.99932	0.35777
125	C	42	35	23	1.434936	96.00662	-103.18	-6.11343	-3.37296	-0.80769
126	H	125	42	35	1.099267	109.5621	-37.6631	-5.64764	-2.93106	0.084577
127	C	29	25	23	1.432739	57.99792	-38.6995	-5.71101	-2.46252	-1.99881
128	H	127	29	25	1.097508	110.3592	-89.3077	-6.36233	-2.64124	-2.86389
129	C	25	23	21	1.43152	60.22333	-48.7404	-4.25486	-2.72041	-2.38314
130	H	129	25	23	1.093679	108.9614	92.5313	-3.61867	-2.41059	-1.54923
131	C	23	21	19	1.419235	110.1716	-125.522	-4.03431	-4.21571	-2.59945
132	H	131	23	21	1.099609	109.5883	-114.981	-4.76018	-4.60433	-3.3283
133	C	35	23	21	1.40644	56.85311	-106.045	-4.30003	-4.91614	-1.25626
134	H	133	35	23	1.092888	111.1609	94.25363	-4.15086	-5.99654	-1.3262
135	C	45	36	29	1.417166	56.23294	-32.2097	-7.13208	2.115616	0.675384
136	H	135	45	36	1.100839	111.4675	-89.9664	-7.58849	1.372409	1.347084
137	H	135	45	36	1.093792	106.8085	152.2748	-6.45099	2.733426	1.267676
138	C	36	29	25	1.44295	93.9105	-89.7754	-6.34733	1.375122	-0.40369
139	H	138	36	29	1.097705	109.046	-31.8589	-5.5979	0.729705	0.072493
140	C	30	1	3	1.437431	56.97492	107.3583	-5.63911	2.32565	-1.39311
141	H	140	30	1	1.094418	109.961	-89.5584	-6.35779	3.067934	-1.75402
142	C	1	140	30	1.425872	36.16008	120.2815	-5.08676	1.540578	-2.57526
143	H	142	1	140	1.097707	108.9921	-119.36	-4.2906	0.875375	-2.21666
144	C	27	1	142	1.41336	59.96313	31.6342	-6.18354	0.677677	-3.20441
145	H	144	27	1	1.096756	105.8516	90.83919	-6.98545	1.333202	-3.56511
146	C	36	29	25	1.417032	36.01699	38.55345	-6.78731	-0.24128	-2.13492
147	H	146	36	29	1.091042	106.0071	118.6865	-7.62425	-0.82381	-2.52295

148	C	13	11	9	4.748961	92.63619	66.31279	5.814967	-2.4014	-2.98783
149	C	148	13	11	1.396174	90.47423	84.95754	7.200768	-2.2317	-2.98018
150	C	148	13	11	1.403004	50.69651	-45.9722	4.960947	-1.35658	-2.60387
151	C	149	148	13	1.419133	120.3464	-42.5594	7.769587	-0.99623	-2.5752
152	C	151	149	148	1.402077	118.86	-0.34621	6.916422	0.04823	-2.19175
153	C	152	151	149	1.392889	119.9543	-0.24772	5.536037	-0.13616	-2.21781
154	C	150	148	13	1.465142	119.4513	-116.875	3.512669	-1.57785	-2.59077
155	S	154	150	148	1.760074	122.2777	-178.66	2.372048	-0.31006	-2.1554
156	C	155	154	150	1.75251	88.76543	-179.496	1.066065	-1.45168	-2.40519
157	N	154	150	148	1.307671	123.8606	1.809548	2.947259	-2.71908	-2.88728
158	C	157	154	150	1.373972	112.8076	179.6556	1.577149	-2.67468	-2.7944
159	C	156	155	154	1.482766	120.3532	179.8852	-0.34209	-1.0366	-2.19688
160	N	159	156	155	1.36181	114.4099	-35.8064	-0.52396	-0.1568	-1.17345
161	C	160	159	156	1.446789	123.2824	-177.375	-1.82409	0.331047	-0.76742
162	C	161	160	159	1.536968	108.6928	143.316	-1.83955	0.477368	0.762489
163	N	162	161	160	1.351616	114.9064	168.8514	-2.90492	1.1441	1.259801
164	O	162	161	160	1.232266	120.733	-12.0496	-0.93882	-0.0062	1.450479
165	O	159	156	155	1.240282	122.1846	142.2096	-1.263	-1.43493	-2.92597
166	C	163	162	161	1.463886	122.6324	174.0203	-3.15657	1.270789	2.69632
167	C	166	163	162	1.534489	113.3207	-93.2572	-4.08258	0.179339	3.249404
168	C	167	166	163	1.534302	112.6152	179.7574	-4.33053	0.323982	4.756613
169	C	168	167	166	1.532458	112.5494	179.7785	-5.25979	-0.76355	5.306338
170	O	151	149	148	1.345067	116.084	179.6796	9.112844	-0.92912	-2.5942
171	C	170	151	149	1.435888	119.8133	-177.864	9.774087	0.267827	-2.15619
172	C	171	170	151	1.527613	108.1757	178.4763	11.28181	0.041609	-2.25204
173	C	172	171	170	1.535761	109.3915	175.2566	12.01612	1.344533	-1.90312
174	C	172	171	170	1.53438	111.6292	-60.4675	11.73639	-1.12418	-1.36401
175	C	158	157	154	1.497291	118.2363	-177.125	0.808965	-3.93531	-3.04457
176	C	149	148	13	1.432424	120.1749	136.9816	8.06018	-3.30847	-3.37235
177	N	176	149	148	1.16303	179.2258	-2.21926	8.745369	-4.19193	-3.69276
178	H	148	13	11	1.084038	128.8084	-144.706	5.38781	-3.35167	-3.28728
179	H	152	151	149	1.083213	121.1852	179.7984	7.310087	1.005713	-1.873
180	H	153	152	151	1.085866	117.7513	-179.446	4.908648	0.699431	-1.92238
181	H	161	160	159	1.097711	111.3986	-94.4652	-2.0619	1.287437	-1.25088
182	H	161	160	159	1.095412	109.8749	24.31019	-2.59915	-0.3847	-1.06225
183	H	163	162	161	1.017317	119.016	-9.65031	-3.53016	1.624735	0.617145
184	H	166	163	162	1.093741	107.3649	28.63424	-2.18736	1.235031	3.20191
185	H	166	163	162	1.09618	107.4057	144.5795	-3.59139	2.262486	2.866844
186	H	167	166	163	1.100105	109.1255	-58.4282	-5.04295	0.216122	2.714084
187	H	167	166	163	1.096791	109.4468	58.08591	-3.64574	-0.80645	3.048542
188	H	168	167	166	1.099289	108.9877	-58.7479	-3.36675	0.279813	5.283506
189	H	168	167	166	1.100184	109.6353	57.12531	-4.75121	1.318272	4.968329
190	H	169	168	167	1.095981	111.1627	178.7695	-5.40386	-0.65171	6.387036
191	H	169	168	167	1.097591	110.9656	-61.6108	-6.24898	-0.71063	4.83369
192	H	169	168	167	1.095393	110.7387	58.2446	-4.84699	-1.75986	5.114406
193	H	171	170	151	1.098443	109.1993	-60.7341	9.462413	1.106524	-2.79339
194	H	171	170	151	1.09953	109.2975	57.76483	9.477685	0.488519	-1.12062
195	H	172	171	170	1.098719	106.8857	57.97441	11.50053	-0.21235	-3.29839
196	H	173	172	171	1.095013	110.4467	-177.8	13.09843	1.212552	-2.00432
197	H	173	172	171	1.096721	111.8699	-57.8116	11.7175	2.170319	-2.56016
198	H	173	172	171	1.097672	111.2072	62.95713	11.81638	1.64772	-0.86723
199	H	174	172	171	1.095548	110.5405	178.1372	12.81369	-1.29184	-1.47138
200	H	174	172	171	1.09807	110.9031	-62.4782	11.53539	-0.91194	-0.30556
201	H	174	172	171	1.093521	111.2412	57.98161	11.21949	-2.05022	-1.63057
202	H	175	158	157	1.097364	110.2051	64.51761	1.0483	-4.68339	-2.27821
203	H	175	158	157	1.088885	111.1988	-174.999	-0.2643	-3.75225	-3.02802
204	H	175	158	157	1.095614	109.9041	-53.8746	1.089051	-4.35612	-4.0166
205	H	160	159	156	1.016499	117.7712	-25.7775	0.156978	-0.16083	-0.41875
